# Immune checkpoints expression patterns in early-stage triple-negative breast cancer predict prognosis and remodel the tumor immune microenvironment

**DOI:** 10.3389/fimmu.2023.1073550

**Published:** 2023-02-06

**Authors:** Jinguo Zhang, Hongwei Jin, Shuaikang Pan, Chaoqiang Han, Qingqing Sun, Xinghua Han

**Affiliations:** ^1^ Department of Medical Oncology, Anhui Provincial Hospital Affiliated to Anhui Medical University, Hefei, China; ^2^ Department of Medical Oncology, The First Affiliated Hospital of USTC, Division of Life Science and Medicine, University of Science and Technology of China, Hefei, China; ^3^ School of Medical Oncology, Anhui Medical University, Hefei, China; ^4^ School of Medical Oncology, Wan Nan Medical College, Wuhu, China

**Keywords:** triple-negative breast cancer, immune checkpoint genes, molecular subtypes, prognosis, nomogram

## Abstract

**Background:**

Currently, targeting immune checkpoint molecules holds great promise for triple-negative breast cancer (TNBC). However, the expression landscape of immune checkpoint genes (ICGs) in TNBC remains largely unknown.

**Method:**

Herein, we systematically investigated the ICGs expression patterns in 422 TNBC samples. We evaluated the ICGs molecular typing based on the ICGs expression profile and explored the associations between ICGs molecular subtypes and tumor immune characteristics, clinical significance, and response to immune checkpoint inhibitors (ICIs).

**Results:**

Two ICGs clusters and two ICGs-related gene clusters were determined, which were involved in different survival outcomes, biological roles and infiltration levels of immune cells. We established a quantification system ICGs riskscore (named IRS) to assess the ICGs expression patterns for individuals. TNBC patients with lower IRS were characterized by increased immune cell infiltration, favorable clinical outcomes and high sensitivity to ICIs therapy. We also developed a nomogram model combining clinicopathological variables to predict overall survival in TNBC. Genomic feature analysis revealed that high IRS group presented an increased tumor mutation burden compared with the low IRS group.

**Conclusion:**

Collectively, dissecting the ICGs expression patterns not only provides a new insight into TNBC subtypes but also deepens the understanding of ICGs in the tumor immune microenvironment.

## Introduction

As reported in the GLOBOCAN 2020 survey, the number of breast cancer (BC) has recently overtaken lung cancer as the most common cancer type worldwide and has become the leading cause of cancer death in women (1). It has been reported that triple negative breast cancer (TNBC) makes up approximately 15%-20% of all BC patients, which is characterized by estrogen (ER), progesterone (PR), and human epidermal growth factor receptor-2 (HER2) negativity (2). Due to the variety and heterogeneity, distinct molecular subtypes of TNBC are still not completely understood. As defined by Lehmann et al., the molecular subtypes of TNBC consist of basal-like 1 (BL1), basal-like 2 (BL2), mesenchymal (M) and luminal androgen receptor (LAR) (3). According to the components of the tumor immune microenvironment, TNBC has been divided into four distinct subsets including fully inflamed (FI), stroma restricted (SR), margin-restricted (MR), and immune desert (ID) (4, 5). Different molecular subtypes of TNBC have different etiology, prognosis and clinical-pathological characteristics. Several clinical trials are ongoing to prove the the clinical applicability and effect of subtyping-based targeted therapy for refractory metastatic TNBC (6, 7). Therefore, more efforts are still needed to advance the TNBC subtypes research.

As TNBC lacks established therapeutic targets, surgery and chemotherapy remain the preferred treatment modality (8). However, the efficacy of chemotherapy varies among individuals. Furthermore, TNBC presents a more aggressive phenotype than other BC subtypes and most patients ultimately succumb to tumor recurrence and metastasis (9). In recent years, immunotherapy has emerged as an attractive alternative for conventional cancer treatments (10). TNBC, the most immunogenic subtype of BC, has been regarded as the most suitable candidate for immunotherapy (11). The effect of immunotherapies on early-stage TNBC and metastatic TNBC has recently been studied in several clinical trials (12, 13). However, only a minority of TNBC patients achieve durable clinical benefits from anti-PD-1/L1 treatments (14). Hence, identification of the most suitable subgroups of TNBC patients who can truly benefit from immunotherapy is urgently required.

Immune check-point inhibitors (ICIs) are becoming the most effective immunotherapeutic approaches for cancer treatments (15). ICIs aim to target immune check-point genes (ICGs) to reverse the immunosuppressive tumor environment, and thus enhance the anti-tumor responses by activating infiltrating immune cells (16). Indeed, the efficacy of immunotherapy largely depends on the host tumor microenvironment and immune status (17). Therefore, the development of quantitative biomarkers for individual immune status is warranted. Currently, the ICGs expression patterns have been investigated in lung adenocarcinoma and gastric cancer (18, 19). However, the effect of ICGs patterns on the immune characteristics of TNBC remains unclear. In this study, we explored the ICGs expression profiles and identified distinct ICGs molecular typing in TNBC. We also established a risk scoring system (IRS) to evaluate immune function in individual patients. Our results dissected the role of ICGs expression patterns in molecular subtypes, tumor-infiltrating immune cells and prognosis evaluation.

## Materials and methods

### TNBC dataset and preprocessing

In this study, the transcriptome expression data, and clinical characteristics of TNBC patients were derived from TCGA database (https://cancergenome.nih.gov/) and METABRIC dataset. The METABRIC datasets are derived from the UK-Canada METABRIC project (20). The detail clinical information of TNBC in TCGA cohort and METABRIC cohort was summarized in **Supplementary Table S1**. A total of 422 TNBC tumor samples were enrolled for analysis including the TCGA-TNBC dataset. (N = 123) and METABRIC-TNBC (N = 299). The list of ICGs was acquired according to a previous report (21). The normalized matrix expression and clinical data of METABRIC-TNBC were obtained from the cBioPotal website (http://www.cbioportal.org/) (22). For the TCGA-TNBC data, the values of the RNA sequencing data (FPKM) were converted into transcripts per kilobase million (TPM) values. We obtained somatic mutation profiles from TNBC patients from the TCGA data portal. In addition, TNBC patients who missed corresponding clinical data were excluded from further analyses.

### Construction of ICGs expression clusters by consensus molecular clustering

We combined the transcriptome data of TCGA-TNBC and METABRIC-TNBC into final expression data. Batch effect of individual datasets was removed using “ComBat” in R. The expression of ICGs was extracted for further analysis. To identify distinct ICGs expression patterns, unsupervised clustering analysis was applied based on the expression of ICGs. We established the optimal cluster number based on the cophenetic correlation, dispersion, silhouette, and other factors. Consensus clustering was performed with the package “ConsensusClusterPlus” with 1000 times repetitions (23).

### Gene set variation analysis and immune cell infiltration analysis

To further understand the biological phenomena in different TNBC ICG clusters, the R package “gsva” was used to perform GSVA. We retrieved the gene sets ‘c2.cp.kegg.v7.2.symbols’ and ‘h.all.v7.4.symbols’ from the MSigDB database (http://www.gsea-msigdb.org/gsea/downloads.jsp ). We selected the top 20 biological terms with adjusted P < 0:05. To quantify the enrichment score of 23 immune cells infiltrating in different TNBC ICG clusters, we performed a single-sample gene set enrichment analysis (ssGSEA) (24). The enrichment scores were rescaled to a continuous scale of 0 to 1.

### Generation of ICGs gene signature

Our differentially expressed genes (DEGs) screening among different ICGs clusters was performed using the “limma” R package with the criteria of adjusted P < 0.05. GO and KEGG pathways analyses were applied to evaluate the biological processes in the DEGs, which was achieved with the “ggplot2,” “clusterProfiler,” “org.Hs.eg.db” and “enrichplot” R packages (25, 26). The results were visualized with the top 30 biological terms. Then, we selected prognostic value of DEGs using a univariate Cox regression model. The TNBC ICGs gene clusters were constructed based on the expression of prognostic DEGs.

### Construction of ICGs risk score by LASSO regression

The least absolute shrinkage and selection operator (LASSO) method was applied to evaluate the ICGs expression patterns with a quantization index. The most correlated genes were identified to construct IRS model using the R package “glmnet”. We applied 10-fold cross-validation to optimize the model and reduce overfitting. In this model, a prognostic risk score was computed as follows: IRS= Expression of gene 1 *Coef 1 + Expression of gene 2 *Coef 2 + Expression of gene 3 *Coef 3 + Expression of gene n *Coef n. In our study, we divided the TNBC patients into high- and low-risk subgroups based on the median risk score for subsequent study. Dimension reduction analysis was performed by the techniques of principal component analysis (PCA) and t-distributed stochastic neighbor embedding (t-SNE). A receiver operating characteristic curve (ROC curve) was drawn with the R package “survivalROC” (27). By using the “survival” and “survminer” packages in R, survival curves were plotted for the high- and low-risk groups.

### Establishment of a predictive nomogram and mutation analysis

It has been widely accepted that nomograms predict cancer prognosis (28). A nomogram was constructed by integrating variables that could serve as independent prognostic factors including tumor stage, age, tumor size, node status and IRS model. We calculated a nomogram score to predict the probability of 1-, 3-, and 5 years OS (overall survival) of TNBC. Assessment of the calibration capability of the nomogram was performed by plotting calibration curves. Regarding the effect of IRS on TNBC mutation profiles, different IRS groups of mutations were visualized using the ‘maftools’ package in R.

### Evaluation of immune infiltration characteristics and ICIs therapy

To estimate the immune cells composition in TNBC samples, the CIBERSORT analyses were performed on expression data to calculate the abundance of 22 types of immune cells (29). The Wilcoxon rank-sum test was adopted to detect the difference of 22 immune cells in the high- and low-risk TNBC groups. The estimator, immune, and stromal scores were calculated for TNBC samples based on the ESTIMATE algorithm of the “estimate” package (30). In addition, we further compared the immune function in the high- and low-risk TNBC groups. Analyses of IRS model genes and CD8 T cells infiltration were carried out using the immune gene module in the TIMER2 database (http://timer.comp-genomics.org/) (31). On the basis of mRNA expression, tumor stemness was found to be measured by RNA stemness score (RNAss) and DNA stemness score (DNAss) (32). Correlation analysis between IRS and RNAss or DNAss was tested by Spearman’s rank correlation test. To estimate the immune state of TCGA-TNBC samples, immunophenoscore (IPS) was calculated from an online database (https://tcia.at/) (33). Analysis of IPS in different IRS groups was conducted based on the status of CTLA-4 and PD-1 expression. We predicted chemotherapeutic response in patients using the R package ‘pRRophetic’ (34).

### Statistical analysis

Analyses of all data in this study were performed using R software (version 4.1.1). Comparisons between two groups were conducted using Wilcoxon rank sum tests. We performed Kaplan-Meier and log-rank survival analyses on a univariate basis. The correlations were calculated using Spearman’s rank correlation. *P* values less than 0.05 were considered statistically significant.

## Results

### ICGs expression patterns in TNBC

**Figure 1:**
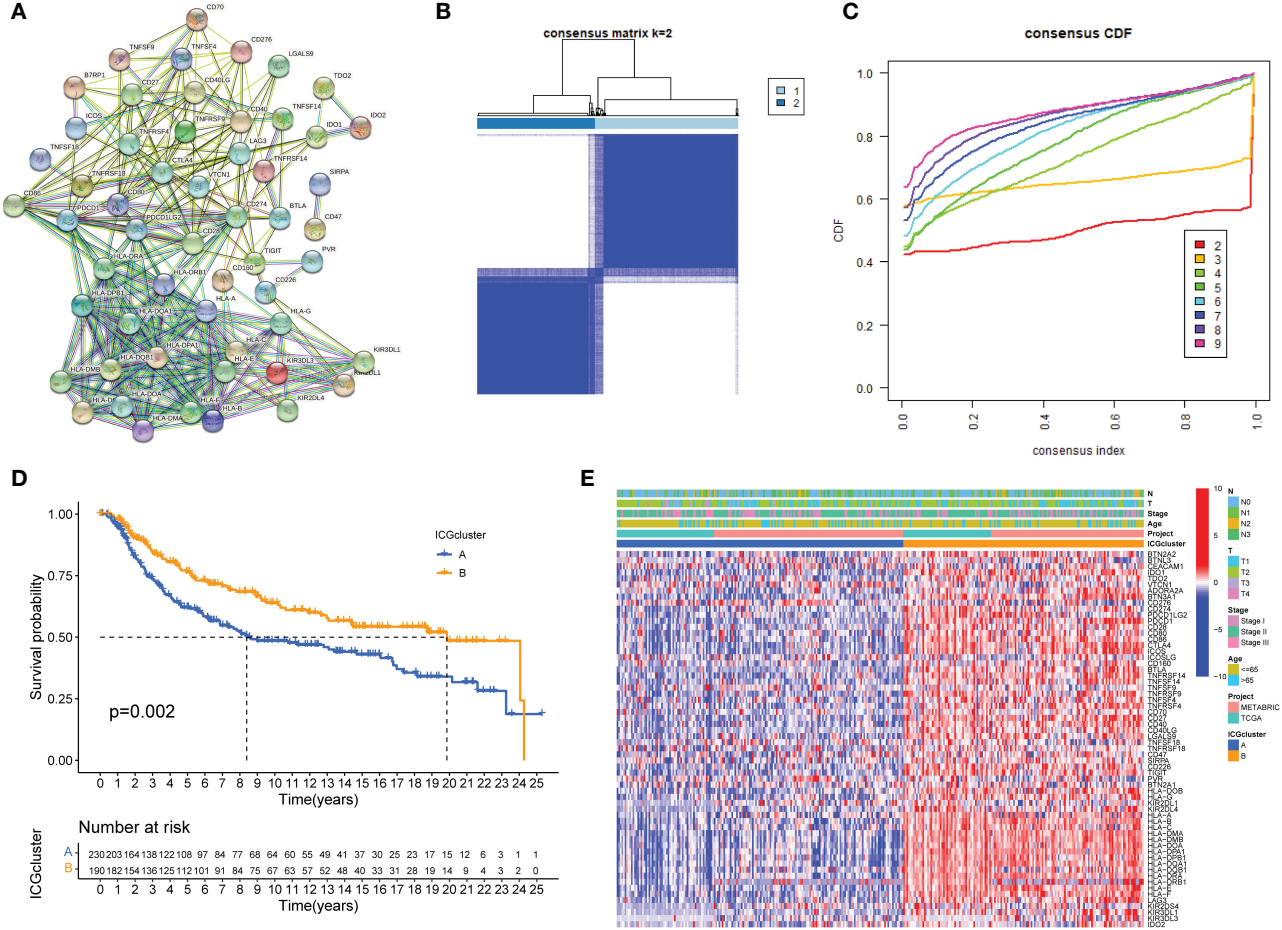
ICGs expression patterns in TNBC. **(A)** Protein interaction network of ICGs by STRING database. **(B, C)** Consensus clustering matrix for k = 3 based on ICG expression. **(D)** Kaplan-Meier curves of OS for two TNBC subtypes. The numbers of patients in ICGscluster-A and ICGscluster-B subtypes are 230 and 190, respectively. **(E)** Unsupervised clustering of ICGs expression to divide TNBC patients into two ICGs subtypes. The ICGclusters, datasets, age, stage and survival status were used as patient annotations.

Typically, solid cancers exploit several mechanisms to escape immune surveillance. Among them, overexpressed ICGs in cancer cells are the key regulators to escape the body’s immune system. It is well recognized that the antitumor response is tightly regulated by checkpoint genes between tumor cells and immune cells (35). A complex and finely tuned signaling network of ICGs was presented by the STRING database (**Figure 1A**). The prognostic value of ICGs in TNBC was evaluated by univariate Cox regression analysis and Kaplan–Meier (KM) log-rank test (**Supplementary Table S2**). We next combined TCGA-TNBC and METABRIC-TNBC datasets to perform consensus clustering analysis. Unsupervised clustering analysis revealed two distinct ICGs clusters in TNBC (**Figure 1B, C**). Interestingly, TNBC patients with ICGscluster-B had a longer survival time than ICGscluster-A (**Figures 1D**). ICGs expression profile analyses found that ICGscluster-B subgroup exhibited higher expression of checkpoint genes compared to ICGscluster-A group (**Figure 1E**).

### Construction of ICGs-related gene clusters in TNBC

**Figure 2:**
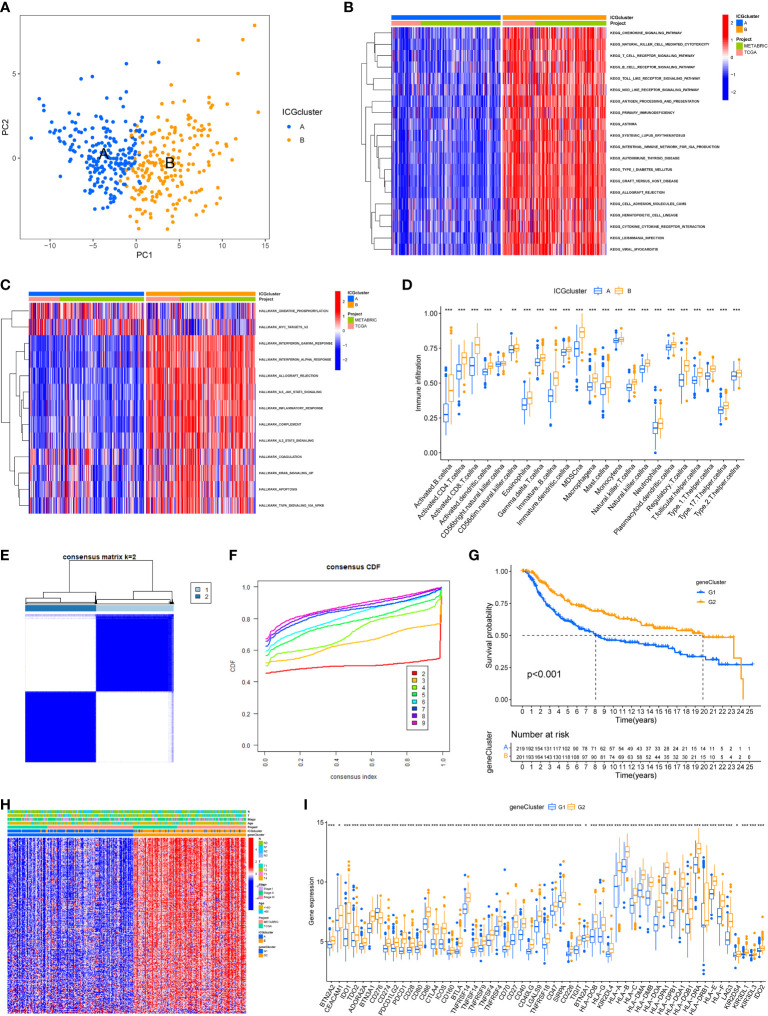
Identification of ICGs-related geneclusters in TNBC. **(A)** Principal component analysis of two immune checkpoint patterns, showing a remarkable difference between different patterns. **(B)** Heat map showed the GSVA score of KEGG pathways in two ICGs subtypes. Red indicated activated pathways, and blue indicated inhibited pathways. **(C)** Heat map showed the GSVA score of Hallmark signature in two ICGs subtypes. Red indicated high enrichment Hallmark. **(D)** The abundance of each infiltrating immune cells in two ICGs clusters. The lines in the boxes indicated the median value. The top and bottom ends of the boxes were interquartile range of values. **(E, F)** The identification of ICGs geneClusters by consensus clustering matrix for k = 2. **(G)** The survival curves of the ICGs geneClusters were plotted by the Kaplan-Meier plotter. The numbers of patients in geneCluster G1 and geneCluster G2 subtypes are 219 and 201. **(H)** Unsupervised clustering of prognostic ICGs-related DEGs to divide patients into two genomic subtypes. The ICGs geneClusters, ICGs clusters, datasets, age stage and survival status were used as patient annotations. **(I)** The expression of ICGs in two ICGs geneClusters (*P < 0.05; **P < 0.01; ***P < 0.001).

Principal component analysis was conducted to classify TNBC samples into two classes with clear differences after dimensionality reduction (**Figure 2A**). To further explore the biological behaviors between different ICGs cluster, the results of GSVA revealed that ICGscluster-B was highly enriched in various immune-related pathways, such as natural killer (NK) cell mediated cytotoxicity, T cell receptor signaling pathway, interferon gamma response and IL-6 JAK STAT3 signaling (**Figures 2B, C**). To further characterize the immune infiltration in different ICGs clusters, we applied the ssGSEA method in TNBC samples. As shown in **Figure 2D**, ICGscluster-B patients had a higher abundance of antitumor immune cells including activated CD8 T cell, activated dendritic cell and so on. We next identified ICGs-related DEGs using the “limma” algorithm and 570 DEGs were recognized between two ICGs clusters (**Supplementary Table S3**). GO and KEGG enrichment analyses displayed that these ICGs-related DEGs were mainly involved in T cell activation, MHC protein complexs, immune receptor activity and cytokine−cytokine receptor interactions (**Supplementary Figures S1** and **S2**). The univariate cox regression analysis was applied to screen the prognostic ICGs-related DEGs (**Supplementary Table S4**). We next conducted the consensus clustering analysis and the results showed two ICGs geneClusters (named ICGs-G1 and ICGs-G2) based on the expression of prognostic ICGs-related DEGs (**Figures 2E, F**). Survival analysis indicated that patients with ICGs-G2 were associated with better prognosis (**Figure 2G**). The heat plot showed that ICGs-G2 patients were largely overlapped with ICGscluster-B subtype (**Figure 2H**). As expected, higher expression of ICGs was observed in ICGs-G2 subtype compared to ICGs-G1 subgroup (**Figure 2I**).

### Generation of an ICGs risk model

**Figure 3:**
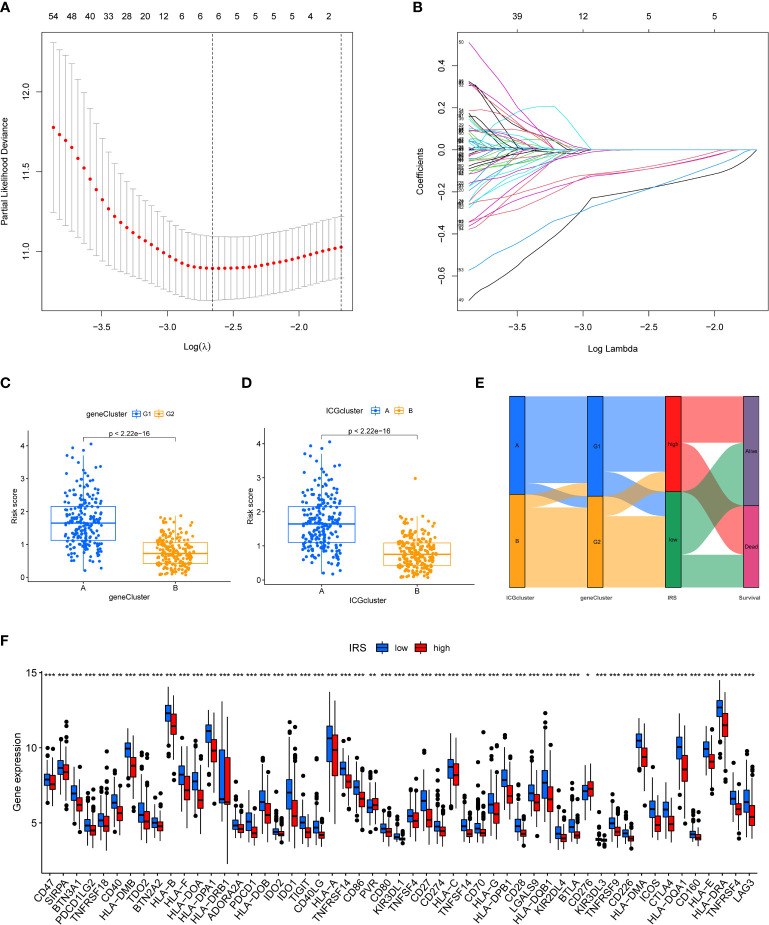
Construction of ICGs-related risk model. **(A)** Elucidation for LASSO coefficient profiles of prognostic ICGs. **(B)** The least absolute shrinkage was performed and construction of selection operator (LASSO) regression model. **(C)** The correlation between IRS model and ICGs geneClusters. **(D)** The correlation between IRS model and ICGs cluster. **(E)** Alluvial diagram of ICGs clusters in groups with different geneClusters, IRS, and survival status. **(F)** The expression of ICGs in high and low IRS group (*P < 0.05; **P < 0.01; ***P < 0.001).

We next constructed a Lasso logistic regression model to assess the immune status for individual TNBC patients. Four ICGs-related genes were selected to generate the best risk score model (named IRS) (**Figures 3A, B**). The riskscore was calculated by the formula IRS = expression level of FGD3 *(-0.22) + expression level of TMEM176A*(-0.19) + expression level of CD1B*(-0.57) + expression level of MATK*(-0.24). We also noted that TNBC patients with ICGs-G2 subtype or ICGscluster-B subtype had a lower IRS, indicating that IRS was a poor prognostic indicator (**Figures 3C, D**). The relationships among ICGs clusters, ICGs geneCluster and IRS were illustrated as **Figure 3E**. The ICGscluster-B subtype in ICGs-G2 subpopulation was linked to a low IRS. Finally, we found TNBC patients with a low IRS had higher expression of ICGs than those with a high IRS (**Figure 3F**).

### Validation of the efficacy of ICGs-related riskscore model

**Figure 4:**
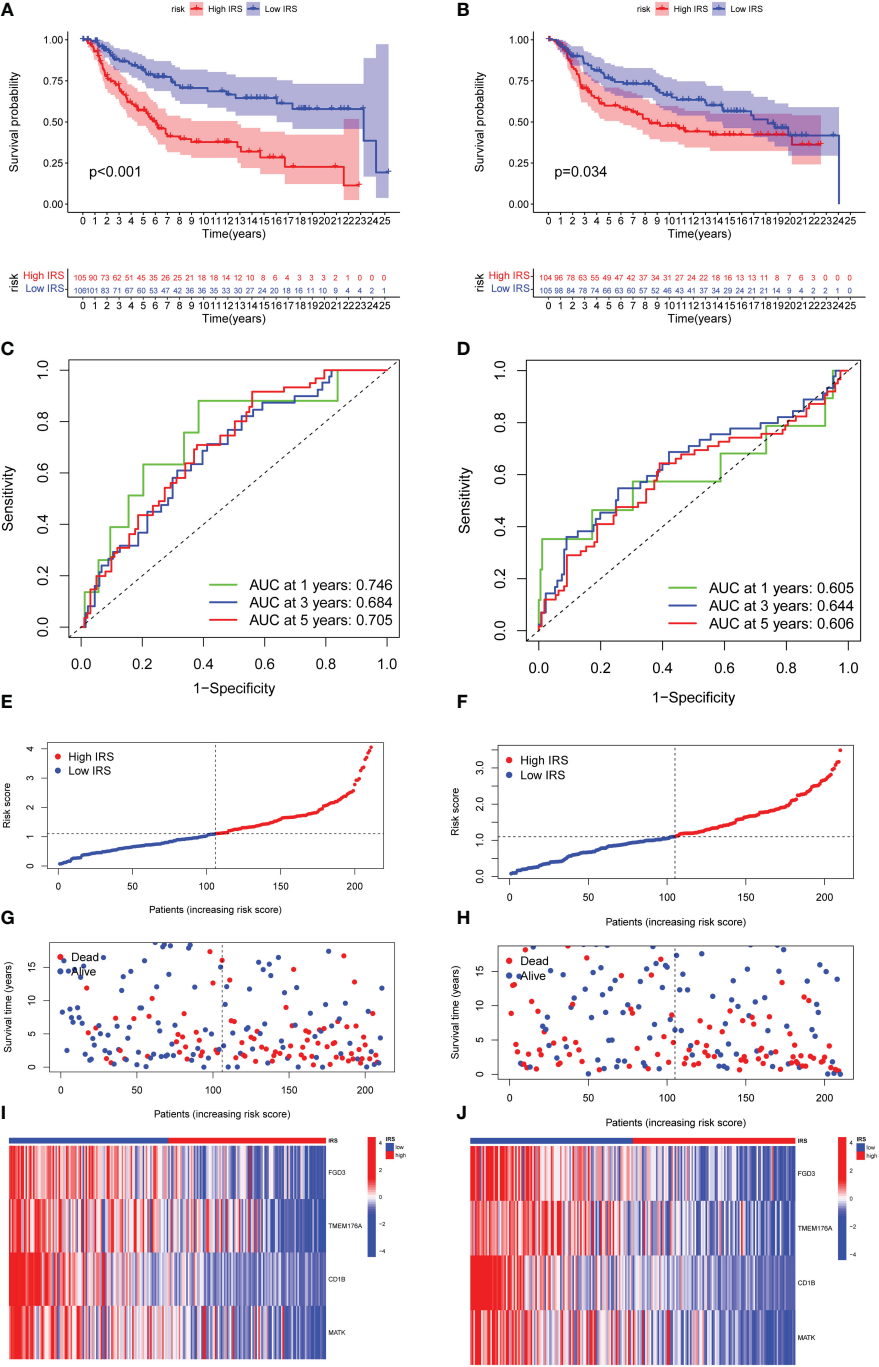
Validation of the efficacy of ICGs-related riskscore model. **(A, B)** Kaplan–Meier curves for OS in TNBC patients according to the riskscore stratification in the training cohort and validation cohort. **(C, D)** Time-dependent ROC analysis for OS prediction at 1 year, 3 year and 5 year in TNBC patients in the training cohort and validation cohort. **(E, F)** Distribution of risk score for the training group and validation cohort. **(G, H)** Distribution of survival time of patients in the training and validation cohort. **(I, J)** Heatmap depicting the expression of FGD3, TMEM176A, CD1B and MATK between high IRSgroup and the low IRSgroup in training and validation group.

We then divided the TNBC cohort into a training cohort (N = 211) and a validation cohort (N = 209). The clinical characteristics of two cohorts were summarized in **Supplementary Table S5**. Patients were classified into low-risk and high-risk groups based on their median risk scores. In both the training and validation cohort, TNBC patients with high IRS predicted shorter OS than those with low IRS (**Figures 4A, B**). Time-dependent ROC curves also exhibited excellent stability of our IRS model in the training cohort (5‐year AUC, 0.705; 3‐year AUC, 0.684; 1‐year AUC, 0.746; **Figure 4C**) and validation cohort (5‐year AUC, 0.606; 3‐year AUC, 0.644; 1‐year AUC, 0.605; **Figure 4D**). The distribution of IRS model in training cohort and validation cohort was presented in **Figures 4E, F**. Moreover, the scatter plot showed that the mortality rate of TNBC patients increased with the increased level of IRS in both the training cohort and validation cohort (**Figures 4G, H**). As shown in **Figures 4I, J**, high expression of FGD3, TMEM176A and MATK was observed in low IRS group.

### Construction of nomogram for predicting OS

**Figure 5:**
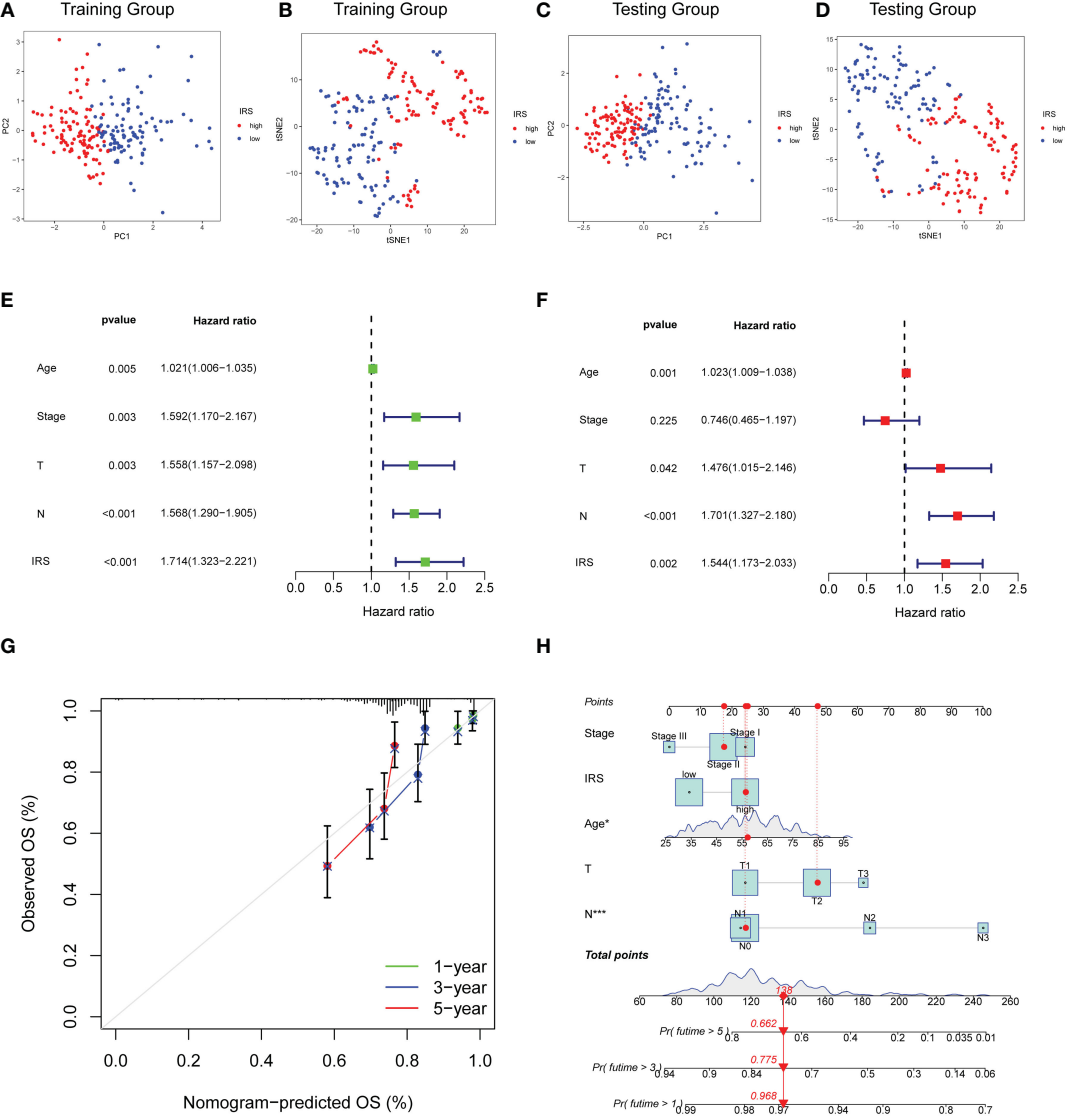
Construction of Nomogram for predicting OS in TNBC. **(A, B)** PCA analysis and t-SNE analysis of TNBC patients based on IRS model for the training cohort. **(C, D)** PCA analysis and t-SNE analysis of TNBC patients based on IRS model for the validation cohort. **(E)** Forest plot for IRS and clinical features in TNBC patients by univariate analyses. **(F)** The multivariate Cox forest plot of IRS and clinical characteristics. **(G)** Calibration curves for predicting patients’ OS at 1-, 3-, and 5-year in TNBC patients based on the nomogram. **(H)** Nomogram depending on the IRS and other clinicopathologic feature predicting the 1-, 3-, and 5-year overall survival for TNBC patients.

PCA and t-SNE methods were applied to separate two risk subgroups. As shown in **Figures 5A–D**, different risk subgroups were divided into two obvious directions. To explore whether the effect of our IRS model was an independent factor, we combined clinicopathological variables including age, stage tumor size and node status with IRS to perform Cox analyses. Univariable Cox regression analysis showed that IRS was a high-risk factor for TNBC patients [HR =1.714, 95% CI (1.323, 2.221), p < 0.001, **Figure 5E**]. Multivariate Cox analysis further supported that IRS could serve as an adverse independent prognostic factor [HR=1.544, 95% CI (1.173, 2.003), p =0.002, **Figure 5F**]. We also performed multivariable Cox-regression analysis adjusting for stage, IRS and the infiltration of immune cells. The results further confirmed the independent prognostic role of IRS (**Supplementary Figure S3**). We next developed a nomogram combining clinicopathological features and IRS model to help clinicians to predict the survival of TNBC patients. Calibration curves for OS prediction at 1, 3, and 5 years were as shown in **Figure 5G**. In addition, if the total nomogram score of one patient was 138 points, the estimated survival probabilities for this patient were 96.8%, 77.5%, and 66.2% for 1, 3, and 5 years, respectively (**Figure 5H**).

### Immune characteristics and genomic features in different risk subgroups

**Figure 6:**
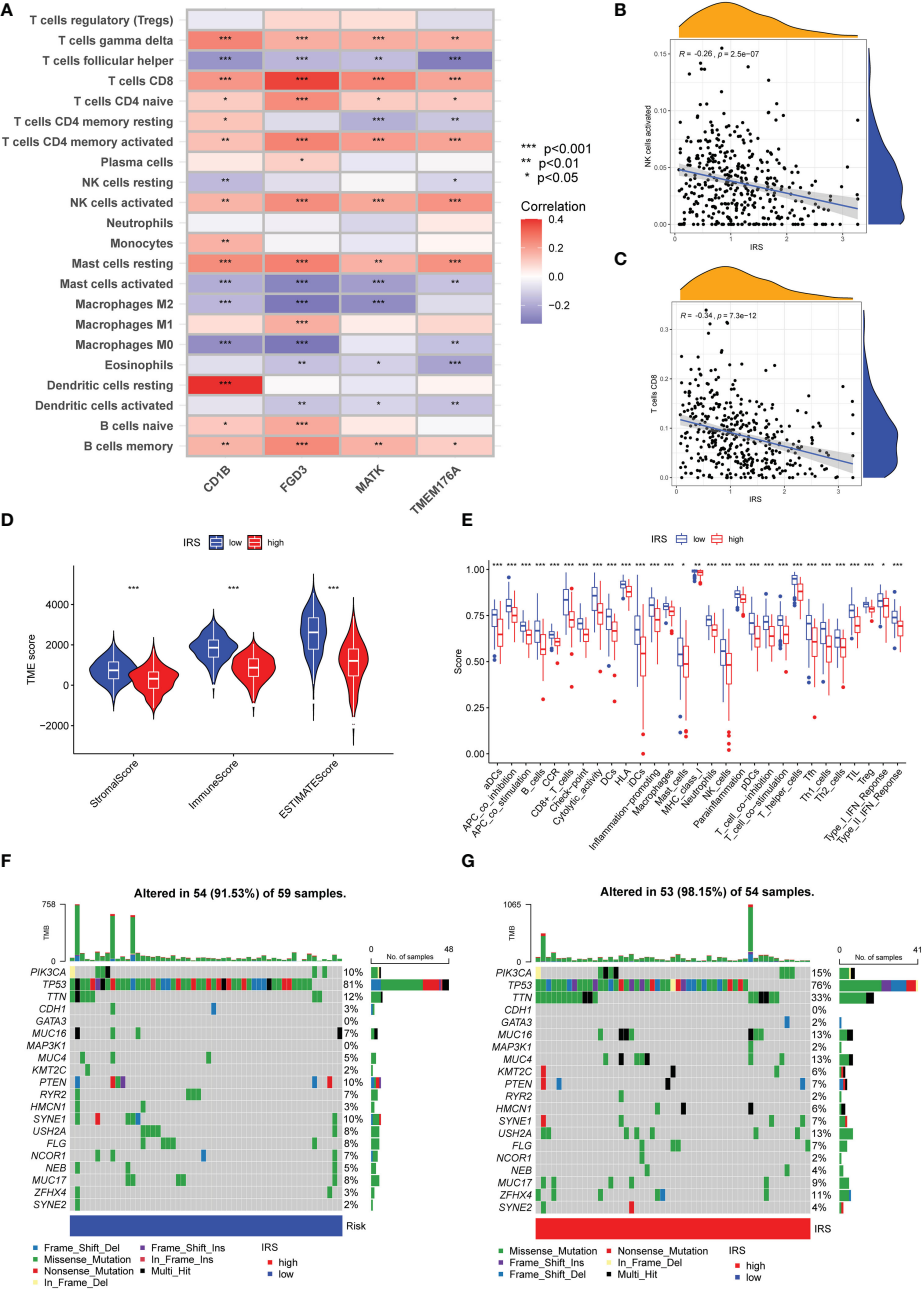
Immune Characteristics and genomic features of ICGs-related riskscore model. **(A)** Heatmap depicting the correlations between the expression of FGD3, TMEM176A, CD1B, MATK and 22 immune infiltration cells. **(B)** Correlation analysis between IRS and the level of activated NK cells infiltration. **(C)** Correlation analysis between IRS and the level of CD8 T cells infiltration. **(D)** Following the ESTIMATE algorithm, the level of stromal, immune, and ESTIMATE scores in different IRS groups. **(E)** Immune function analysis in different IRS groups. **(F**, **G)** The waterfall plot of showed the somatic mutation rate in low IRS and high IRS group. Each column indicated an individual sample. The upper barplot was the tumor mutation burden for an individual sample. The right histogram generalized the percentage of each variant type. The number on the right indicated the mutation frequency for each gene.

We further investigated the immune cells infiltration in different risk subgroups by CIBERSORT algorithm. As shown in **Figure 6A**, the expression of CD1B was strongly associated with the infiltration of CD8 T cells, T help cells and resting dendritic cells. FBP1 had a strong correlation with infiltration of CD8 T cells, memory CD4 T cells and M1 macrophages. Furthermore, a strong positive correlation was observed between the infiltration of CD8 T cells, memory CD4 T cells and activated NK cells. Overall, IRS was negatively correlated with the infiltration of activated NK cells and CD8 T cells (**Figure 6B, C**). M0 macrophages and M2 macrophage cells infiltration were positively correlated with IRS. However, M1 macrophage cells infiltration was negatively correlated with IRS (**Supplementary Figure S4**). We also calculated the relationship between CD8 T cells infiltration and IRS model genes using other algorithms (**Supplementary Figure S5**). The results of the ESTIMATE algorithm also demonstrated that TNBC patients with a low IRS had a higher ImmuneScore and ESTIMATEScore (**Figure 6D**). In addition, the immune function in low IRS patients might be attributed to the activation of various immune cells (**Figure 6E**). High IRS was proven to be positively associated with the stemness index DNAss and RNAss (**Supplementary Figure S6**). Then, we investigated the genomic features in different IRS subgroups of TCGA-TNBC cohort. The waterfall plot demonstrated that high IRS group presented an increased tumor mutation burden compared with the low IRS group (**Figures 6F**, **6G**). TTN (33% vs.12%) had higher mutation rates in high IRS group.

### Predictive value of ICGs-related riskscore model in ICIs therapy

**Figure 7:**
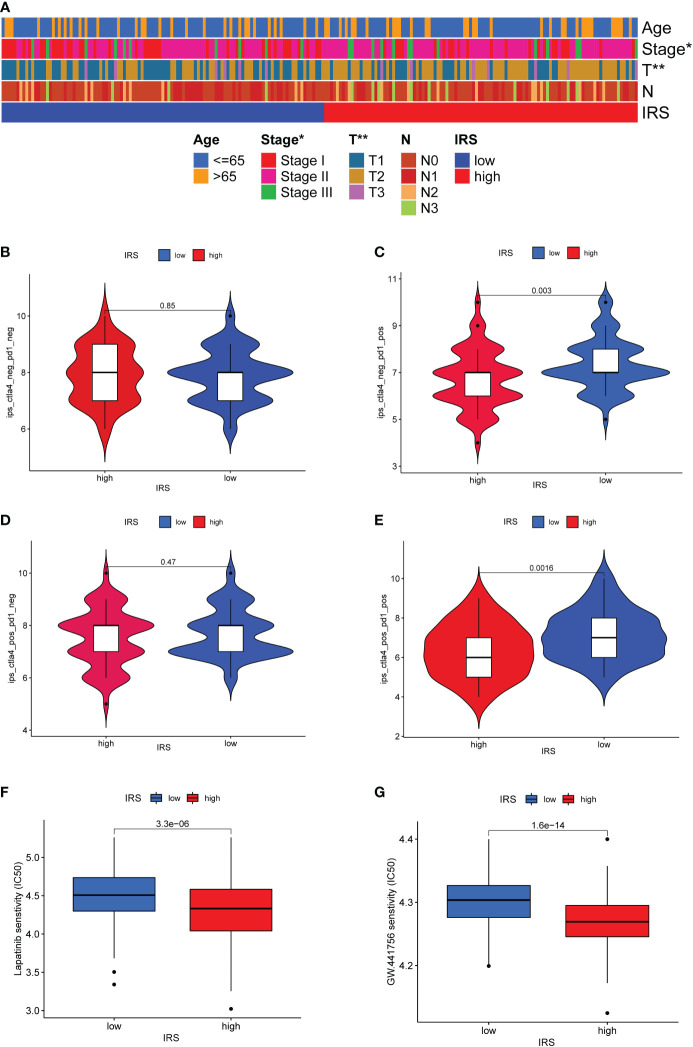
Predictive value of ICGs-related riskscore model in ICIs therapy. **(A)** Heatmap showing the relationship between IRS and clinicopathological parameter. **(B–E)** The relative distribution of IPS identified by the status of CTLA-4 or PD-1 was compared between high IRS group versus low IRS group in TCGA-TNBC cohort. **(F, G)** Drug sensitivity analysis in different IRS groups.

Associations of clinical parameters and the IRS model were also explored. As the **Figure 7A** shown, our IRS model was significantly correlated with tumor stage and tumor size. We next adopted the IPS score to assess the potential clinical efficacy of ICIs in different IRS subgroups. The IPS score is a valid indicator as a measure of immunogenicity. There was no significant difference for TNBC patients with PD-1 negative expression (**Figures 7B, D**). More remarkably, low IRS might serve as a valid candidate biomarker for ICIs in patients with PD-1 positive expression (**Figures 7C, E**). Although TNBC patients with a high IRS might not be the most suitable candidates for ICIs therapy, drug sensitivity analysis revealed that TNBC patients with a high IRS were more sensitive to several targeted drugs or small molecule inhibitors such as lapatinib, GW.441756, A.443654, BIBW2992, Bicalutamide, GNF. 2, NSC.87877 and PF.4708671 (**Figures 7F, G** and **Supplementary Figure S7**).

## Discussion

Immune checkpoint blockade (ICB), an emerging immunotherapy, has been approved to treat multiple cancers (36–38). Although immunotherapy has been widely used in clinical practice, immune-cold cancers still lack a positive clinical outcome (5). TNBC are believed to be more immunogenic than other BC subtypes, but TNBC patients often show heterogeneous responses to ICB therapy. Therefore, the identification of suitable TNBC populations and effective therapeutic targets will develop a novel strategy to improve anti-tumor immune activities. Our study aimed to depict the immune checkpoints expression patterns from a molecular subtyping viewpoint and explore the immunotherapeutic value of ICGs patterns in TNBC. Here, we first investigated the expression of ICGs and evaluated the prognostic value of ICGs in TNBC. Then, ICGs-related subtypes were identified based on the expression of ICGs and ICG-related DEGs. Finally, we constructed a quantitative scoring system IRS and investigated its role in immune cell infiltrates and ICB treatment.

In this study, we first extracted the expression of 53 ICGs from transcriptome data of TCGA-TNBC and depicted the interaction network of ICGs. Inhibitory checkpoint molecules often function as immune inhibitory signaling in the immune system (39). The levels of inhibitory checkpoint molecules have been reported to be involved in predicting patient response to immunotherapy (40). We next revealed two ICG subtypes that were associated with patients’ survival outcomes and immune cells infiltration. Here, our ICGs subtypes were identified based on the expression of ICGs in TNBC. However, extensive studies have revealed the heterogeneity of molecular subtypes of TNBC. Gruosso et al. proposed that TNBC could be classified into four phenotypes: fully inflamed, stroma restricted, margin-restricted, and immune desert (4). Jiang et al. defined TNBC tumors into the following four transcriptional subtypes: luminal androgen receptor, immunomodulatory, basal-like immune-suppressed, and mesenchymal-like (41). For the above two molecular typing, subtypes of fully inflamed and immunomodulatory were characterized by high levels of immune genes or pathways, and satisfactory clinical outcomes. Our results showed that TNBC patients with ICGscluster-B often presented longer OS and higher expression of ICGs than those with ICGsclusters-A.

We further compared transcriptome differences between two ICGs clusters and identified two ICG-related gene subgroups. Considering the individual heterogeneity of TNBC, we established a quantification system IRS to assess the ICGs expression patterns for individuals. Oncotype DX, MammaPrint, and EndoPred are the promising multigene signatures currently available to diagnose recurrence of different BC cancer subtypes (42). So far, no clinical signatures have yet been made to evaluate the immune response in BC. Nevertheless, some gene signatures involved in the immune response in BC have been identified. Tumor-infiltrating lymphocytes (TILs) are the most widely studied immune cells in this context. A large, randomized trial have shown that increasing TIL levels in primary biopsies is associated with better OS and fewer recurrences (43). In addition, Yang et al. identified an immune-enhanced type of BC using 17-gene immune signature, which was associated with improved patient outcomes (44). Our analyses found that lower IRS was correlated with a favorable outcome, indicating the IRS signature was a negative indicator in TNBC. To further verify the accuracy and effectiveness of our IRS model, we compared the survival outcomes, ROC values and risk curves in the training and test cohort. The validation and training sets showed little variation in these parameters, suggesting that our IRS signature has good prediction performance, universality, and accuracy.

In this study, FGD3, TMEM176A, CD1B and MATK were applied to construct our ICGs-related Riskscore. The Facio-Genital Dysplasia 3 gene (FGD3) has been identified as a guanine nucleotide exchange factor for cell division control protein 42 (45). Guo et al. reported that FGD3 inhibited the malignant biological behavior of pancreatic cancer cell by FGD3/HSF4/p65 signaling axis (46). Low expression of FGD3 was an independent poor prognostic factor for overall survival and disease-free survival in young BC patients (47). TMEM176A, located on chromosome 7, belongs to the membrane-spanning 4A gene family (48). Human TMEM176A was initially discovered as a candidate in a screen looking for tumor-associated antigens in human hepatocellular carcinoma (HCC) (49). Recently, the epigenetic regulation of TMEM176A has been uncovered in cancer. The study of Li et al. revealed that reduced expression of TMEM176A in HCC was caused by hypermethylation of the promoter region, which might serve as a novel diagnostic and prognostic marker (50). In human lung cancer, it was reported that TMEM176A potently inhibited the growth of lung cancer cells both *in vitro* and *in vivo* by inhibiting ERK signaling (11). CD1B plays a critical role in regulating the T cells immune response to self- and foreign-lipid antigens (51). The genetic variation of CD1B correlated with biochemical recurrence in patients with localized prostate cancer (52). An increasing body of bioinformatics analyses has identified CD1B as prognostic biomarker in colon cancer and lung cancer (53–56). MATK has been found to phosphorylate and inhibit Src family kinases, and play an inhibitory role in cell growth and proliferation of T-cells (57). Colorectal cancer often exhibits epigenetic inactivation of CHK/MATK, which might promote proliferation and invasiveness through SFK-dependent and independent mechanisms (58). However, the role of FGD3, TMEM176A, CD1B and MATK in anti‐tumor immunity remains unclear.

Previous studies indicated that baseline infiltrating lymphocytes (TILs) could provide independent prognostic value in patients with TNBC in the absence of ICIs therapies or adjuvant chemotherapy (59, 60). A high number of TILs predicts favorable prognosis and pathological complete response in TNBC and HER2-enriched BC subtypes (61). TNBC patients with sparse or unresponsive tumor-infiltrating lymphocytes have limited efficacy of ICIs treatment (62). In the present study, patients with low IRS tended to have a higher infiltrating of CD8 T cells, activated NK cells and M1 Macrophages, which indicated that these patients had a better long-term survival than patients with high IRS and might be more suitable for ICI therapies. Regarding the effect of IRS on mutation burden in TNBC, we found that the low IRS subgroup had a lower mutation rate than the high IRS subgroup. The role of tumor mutation burden (TMB) in TNBC is controversial. The prevailing view is that high TMB patients exhibit a better survival outcome and higher immune cell infiltration than low TMB patients (63). However, some studies reported that TMB in TNBC showed no significant associations with immune cell infiltration (64, 65).

Further analysis revealed that our IRS was related to tumor size and disease stage, which indicated IRS could also serve as an indicator of tumor progression in TNBC. Finally, we verified the predictive value of the IRS model by IPS predictor. A low IRS could be suitable as an indicator for efficiency of immunotherapy in the TCGA-TNBC cohort, especially in those with PD-1 positive expression. Drug sensitivity analysis revealed that patients with high IRS were more sensitive to several targeted drugs or small molecule inhibitors. Thus, we hypothesized that TNBC patients with low IRS should receive immunotherapy, and patients with high IRS should not. To our knowledge, this is the first study to define the TNBC subtypes based on the ICGs expression pattern. However, a few limitations of this study must be acknowledged. Our molecular subtyping was constructed based on the expression of ICGs, and the differences and efficiency between ICGs subtypes and other TNBC subtypes should be compared in the future. Next, we defined a four-gene signature to predict the prognosis of TNBC. Additional clinicopathological parameters might need to be incorporated into our model in the follow-up studies. Also, the standardized TILs scoring in TNBC samples is also required to examine relations with our IRS model. Finally, prospective TNBC cohort and real-world TNBC series are clearly warranted to further examine our findings.

## Conclusions

Our study systematically explored the ICGs expression patterns and defined ICGs-related subtypes in TNBC. In clinical application, we developed an ICGs-related riskscore IRS to stratify TNBC into high or low risk subgroups. Our IRS is a robust biomarker that can be used to evaluate the immune infiltration, survival outcomes, tumor progression and immunotherapy response. Our findings not only provide new insight into the subtype of TNBC but also deepen the understanding of ICGs in the tumor immune microenvironment.

## Data availability statement

Publicly available datasets or databases were analyzed in the present study. This data can be found here: TCGA database (https://portal.gdc.cancer.gov) and cBioPortal database (http://www.cbioportal.org/).

## Author contributions

XH and JZ were responsible for the research design. JZ, and HJ were responsible for data collection, analysis and writing the original draft. SP, QS and CH were responsible for manuscript revision and editing. All authors contributed to the article and approved the submitted version.

## Supplementary material

Supplementary Figure 1 Functional enrichment for ICG-related genes using GO annotation analysis.

Supplementary Figure 2 Functional enrichment for ICG-related genes using KEGG annotation analysis.

Supplementary Figure 3 Multivariable Cox-regression analysis adjusting for stage, IRS and the infiltration of immune cells.

Supplementary Figure 4 Correlation analysis between ICGs-related riskscore and the level of M0 macrophages, M1 macrophages and M2 macrophages.

Supplementary Figure 5 The relationship between CD8 T cells infiltration and IRS model genes by other immune-cell infiltration algorithms.

Supplementary Figure 6 Correlation analysis between IRS and the stemness index DNAss and RNAss.

Supplementary Figure 7 Drug sensitivity analysis in different IRS groups.

## References

[1] Sung H Ferlay J Siegel RL Laversanne M Soerjomataram I Jemal A. Global cancer statistics 2020: GLOBOCAN estimates of incidence and mortality worldwide for 36 cancers in 185 countries. CA: Cancer J Clin (2021) 71(3):209–49. doi: 10.3322/caac.21660 33538338

[2] Huppert LA Gumusay O Rugo HS. Emerging treatment strategies for metastatic triple-negative breast cancer. Ther Adv Med Oncol (2022) 14:17588359221086916. doi: 10.1177/17588359221086916 35422881 PMC9003656

[3] Lehmann BD Bauer JA Chen X Sanders ME Chakravarthy AB Shyr Y. Identification of human triple-negative breast cancer subtypes and preclinical models for selection of targeted therapies. J Clin Invest (2011) 121(7):2750–67. doi: 10.1172/jci45014 PMC3127435 21633166

[4] Gruosso T Gigoux M Manem VSK Bertos N Zuo D Perlitch I. Spatially distinct tumor immune microenvironments stratify triple-negative breast cancers. J Clin Invest (2019) 129(4):1785–800. doi: 10.1172/jci96313 PMC6436884 30753167

[5] Song X Zhou Z Li H Xue Y Lu X Bahar I. Pharmacologic suppression of B7-H4 glycosylation restores antitumor immunity in immune-cold breast cancers. Cancer Discovery (2020) 10(12):1872–93. doi: 10.1158/2159-8290.cd-20-0402 PMC7710601 32938586

[6] Jiang YZ Liu Y Xiao Y Hu X Jiang L Zuo WJ. Molecular subtyping and genomic profiling expand precision medicine in refractory metastatic triple-negative breast cancer: the FUTURE trial. Cell Res (2021) 31(2):178–86. doi: 10.1038/s41422-020-0375-9 PMC8027015 32719455

[7] Wu SY Xu Y Chen L Fan L Ma XY Zhao S. Combined angiogenesis and PD-1 inhibition for immunomodulatory TNBC: concept exploration and biomarker analysis in the FUTURE-C-Plus trial. Mol Cancer (2022) 21(1):84. doi: 10.1186/s12943-022-01536-6 35337339 PMC8951705

[8] Derakhshan F Reis-Filho JS. Pathogenesis of triple-negative breast cancer. Annu Rev Pathol (2022) 17:181–204. doi: 10.1146/annurev-pathol-042420-093238 35073169 PMC9231507

[9] Bianchini G De Angelis C Licata L Gianni L. Treatment landscape of triple-negative breast cancer - expanded options, evolving needs. Nat Rev Clin Oncol (2022) 19(2):91–113. doi: 10.1038/s41571-021-00565-2 34754128

[10] Wang F Cheng F Zheng F. Stem cell like memory T cells: A new paradigm in cancer immunotherapy. Clin Immunol (Orlando Fla) (2022) 241:109078. doi: 10.1016/j.clim.2022.109078 35840054

[11] Li H Yang W Zhang M He T Zhou F GH J. Methylation of TMEM176A, a key ERK signaling regulator, is a novel synthetic lethality marker of ATM inhibitors in human lung cancer. Epigenomics (2021) 13(17):1403–19. doi: 10.2217/epi-2021-0217 34558311

[12] Schmid P Adams S Rugo HS Schneeweiss A Barrios CH Iwata H. Atezolizumab and nab-paclitaxel in advanced triple-negative breast cancer. New Engl J Med (2018) 379(22):2108–21. doi: 10.1056/NEJMoa1809615 30345906

[13] Schmid P Cortes J Pusztai L McArthur H Kümmel S Bergh J. Pembrolizumab for early triple-negative breast cancer. New Engl J Med (2020) 382(9):810–21. doi: 10.1056/NEJMoa1910549 32101663

[14] Nakhjavani M Shigdar S. Future of PD-1/PD-L1 axis modulation for the treatment of triple-negative breast cancer. Pharmacol Res (2022) 175:106019. doi: 10.1016/j.phrs.2021.106019 34861397

[15] Guirgis HM. The impact of PD-L1 on survival and value of the immune check point inhibitors in non-small-cell lung cancer; proposal, policies and perspective. J immunotherapy Cancer (2018) 6(1):15. doi: 10.1186/s40425-018-0320-3 PMC5819662 29463302

[16] Gaikwad S Agrawal MY Kaushik I Ramachandran S Srivastava SK. Immune checkpoint proteins: Signaling mechanisms and molecular interactions in cancer immunotherapy. Semin Cancer Biol (2022) 86:137–50. doi: 10.1016/j.semcancer.2022.03.014 35341913

[17] Jenkins L Jungwirth U Avgustinova A Iravani M Mills AP Haider S. Cancer-associated fibroblasts suppress CD8+ T cell infiltration and confer resistance to immune checkpoint blockade. Cancer Res (2022) 82(16):2904–17. doi: 10.1158/0008-5472.can-21-4141 PMC9379365 35749591

[18] Zhang J Han X Lin L Chen J Wang F Ding Q. Unraveling the expression patterns of immune checkpoints identifies new subtypes and emerging therapeutic indicators in lung adenocarcinoma. Oxid Med Cell Longevity (2022) 2022:3583985. doi: 10.1155/2022/3583985 PMC8843963 35178154

[19] Zhen Z Shen Z Sun P. Dissecting the role of immune checkpoint regulation patterns in tumor microenvironment and prognosis of gastric cancer. Front Genet (2022) 13:853648. doi: 10.3389/fgene.2022.853648 35518357 PMC9061997

[20] Curtis C Shah SP Chin SF Turashvili G Rueda OM Dunning MJ. The genomic and transcriptomic architecture of 2,000 breast tumours reveals novel subgroups. Nature (2012) 486(7403):346–52. doi: 10.1038/nature10983 PMC3440846 22522925

[21] Hu FF Liu CJ Liu LL Zhang Q Guo AY. Expression profile of immune checkpoint genes and their roles in predicting immunotherapy response. Briefings Bioinf (2021) 22(3). doi: 10.1093/bib/bbaa176 32814346

[22] Cerami E Gao J Dogrusoz U Gross BE Sumer SO Aksoy BA. The cBio cancer genomics portal: an open platform for exploring multidimensional cancer genomics data. Cancer Discovery (2012) 2(5):401–4. doi: 10.1158/2159-8290.cd-12-0095 PMC3956037 22588877

[23] Wilkerson MD Hayes DN. ConsensusClusterPlus: a class discovery tool with confidence assessments and item tracking. Bioinf (Oxford England) (2010) 26(12):1572–3. doi: 10.1093/bioinformatics/btq170 PMC2881355 20427518

[24] Hänzelmann S Castelo R Guinney J. GSVA: gene set variation analysis for microarray and RNA-seq data. BMC Bioinf (2013) 14:7. doi: 10.1186/1471-2105-14-7 PMC3618321 23323831

[25] Yu G. Gene ontology semantic similarity analysis using GOSemSim. Methods Mol Biol (Clifton NJ) (2020) 2117:207–15. doi: 10.1007/978-1-0716-0301-7_11 31960380

[26] Yu G Wang LG Han Y He QY. clusterProfiler: an r package for comparing biological themes among gene clusters. Omics J Integr Biol (2012) 16(5):284–7. doi: 10.1089/omi.2011.0118 PMC3339379 22455463

[27] Heagerty PJ Lumley T Pepe MS. Time-dependent ROC curves for censored survival data and a diagnostic marker. Biometrics (2000) 56(2):337–44. doi: 10.1111/j.0006-341x.2000.00337.x 10877287

[28] Iasonos A Schrag D Raj GV Panageas KS. How to build and interpret a nomogram for cancer prognosis. J Clin Oncol Off J Am Soc Clin Oncol (2008) 26(8):1364–70. doi: 10.1200/jco.2007.12.9791 18323559

[29] Newman AM Liu CL Green MR Gentles AJ Feng W Xu Y. Robust enumeration of cell subsets from tissue expression profiles. Nat Methods (2015) 12(5):453–7. doi: 10.1038/nmeth.3337 PMC4739640 25822800

[30] Yoshihara K Shahmoradgoli M Martínez E Vegesna R Kim H Torres-Garcia W. Inferring tumour purity and stromal and immune cell admixture from expression data. Nat Commun (2013) 4:2612. doi: 10.1038/ncomms3612 24113773 PMC3826632

[31] Li T Fu J Zeng Z Cohen D Li J Chen Q. TIMER2.0 for analysis of tumor-infiltrating immune cells. Nucleic Acids Res (2020) 48(W1):W509–w14. doi: 10.1093/nar/gkaa407 PMC7319575 32442275

[32] Malta TM Sokolov A Gentles AJ Burzykowski T Poisson L Weinstein JN. Machine learning identifies stemness features associated with oncogenic dedifferentiation. Cell (2018) 173(2):338–54.e15. doi: 10.1016/j.cell.2018.03.034 29625051 PMC5902191

[33] Charoentong P Finotello F Angelova M Mayer C Efremova M Rieder D. Pan-cancer immunogenomic analyses reveal genotype-immunophenotype relationships and predictors of response to checkpoint blockade. Cell Rep (2017) 18(1):248–62. doi: 10.1016/j.celrep.2016.12.019 28052254

[34] Geeleher P Cox N Huang RS. pRRophetic: an r package for prediction of clinical chemotherapeutic response from tumor gene expression levels. PloS One (2014) 9(9):e107468. doi: 10.1371/journal.pone.0107468 25229481 PMC4167990

[35] Zou W Chen L. Inhibitory B7-family molecules in the tumour microenvironment. Nat Rev Immunol (2008) 8(6):467–77. doi: 10.1038/nri2326 18500231

[36] Chang JY Verma V Welsh JW Formenti SC. Radiotherapy plus immune checkpoint blockade in PD(L)-1-resistant metastatic NSCLC. Lancet Oncol (2022) 23(4):e156. doi: 10.1016/s1470-2045(22)00134-6 35358456

[37] Hoch T Schulz D Eling N Gómez JM Levesque MP Bodenmiller B. Multiplexed imaging mass cytometry of the chemokine milieus in melanoma characterizes features of the response to immunotherapy. Sci Immunol (2022) 7(70):eabk1692. doi: 10.1126/sciimmunol.abk1692 35363540

[38] Kikuchi H Matsui A Morita S Amoozgar Z Inoue K Ruan Z. Increased CD8+ T-cell infiltration and efficacy for multikinase inhibitors after PD-1 blockade in hepatocellular carcinoma. J Natl Cancer Institute (2022) 114(9):1301–5. doi: 10.1093/jnci/djac051 PMC9468280 35288743

[39] Kim HR Park HJ Son J Lee JG Chung KY Cho NH. Tumor microenvironment dictates regulatory T cell phenotype: Upregulated immune checkpoints reinforce suppressive function. J immunotherapy Cancer (2019) 7(1):339. doi: 10.1186/s40425-019-0785-8 PMC6894345 31801611

[40] Gentles AJ Newman AM Liu CL Bratman SV Feng W Kim D. The prognostic landscape of genes and infiltrating immune cells across human cancers. Nat Med (2015) 21(8):938–45. doi: 10.1038/nm.3909 PMC4852857 26193342

[41] Jiang YZ Ma D Suo C Shi J Xue M Hu X. Genomic and transcriptomic landscape of triple-negative breast cancers: Subtypes and treatment strategies. Cancer Cell (2019) 35(3):428–40.e5. doi: 10.1016/j.ccell.2019.02.001 30853353

[42] Cardoso F Kyriakides S Ohno S Penault-Llorca F Poortmans P Rubio IT. Early breast cancer: ESMO clinical practice guidelines for diagnosis, treatment and follow-up†. Ann Oncol Off J Eur Soc Med Oncol (2019) 30(8):1194–220. doi: 10.1093/annonc/mdz173 31161190

[43] Loi S Sirtaine N Piette F Salgado R Viale G Van Eenoo F. Prognostic and predictive value of tumor-infiltrating lymphocytes in a phase III randomized adjuvant breast cancer trial in node-positive breast cancer comparing the addition of docetaxel to doxorubicin with doxorubicin-based chemotherapy: BIG 02-98. J Clin Oncol Off J Am Soc Clin Oncol (2013) 31(7):860–7. doi: 10.1200/jco.2011.41.0902 23341518

[44] Yang B Chou J Tao Y Wu D Wu X Li X. An assessment of prognostic immunity markers in breast cancer. NPJ Breast Cancer (2018) 4:35. doi: 10.1038/s41523-018-0088-0 30393759 PMC6206135

[45] Hayakawa M Matsushima M Hagiwara H Oshima T Fujino T Ando K. Novel insights into FGD3, a putative GEF for Cdc42, that undergoes SCF(FWD1/beta-TrCP)-mediated proteasomal degradation analogous to that of its homologue FGD1 but regulates cell morphology and motility differently from FGD1. Genes to Cells devoted to Mol Cell Mech (2008) 13(4):329–42. doi: 10.1111/j.1365-2443.2008.01168.x 18363964

[46] Guo F Cheng X Jing B Wu H Jin X. FGD3 binds with HSF4 to suppress p65 expression and inhibit pancreatic cancer progression. Oncogene (2022) 41(6):838–51. doi: 10.1038/s41388-021-02140-6 34975151

[47] Renda I Bianchi S Vezzosi V Nori J Vanzi E Tavella K. Expression of FGD3 gene as prognostic factor in young breast cancer patients. Sci Rep (2019) 9(1):15204. doi: 10.1038/s41598-019-51766-w 31645624 PMC6811624

[48] Cuajungco MP Podevin W Valluri VK Bui Q Nguyen VH Taylor K. Abnormal accumulation of human transmembrane (TMEM)-176A and 176B proteins is associated with cancer pathology. Acta histochemica (2012) 114(7):705–12. doi: 10.1016/j.acthis.2011.12.006 PMC5419829 22244448

[49] Wang Y Han KJ Pang XW Vaughan HA Qu W Dong XY. Large Scale identification of human hepatocellular carcinoma-associated antigens by autoantibodies. J Immunol (Baltimore Md 1950) (2002) 169(2):1102–9. doi: 10.4049/jimmunol.169.2.1102 12097419

[50] Li H Zhang M Linghu E Zhou F Herman JG Hu L. Epigenetic silencing of TMEM176A activates ERK signaling in human hepatocellular carcinoma. Clin Epigenet (2018) 10(1):137. doi: 10.1186/s13148-018-0570-4 PMC6219251 30400968

[51] Beckman EM Porcelli SA Morita CT Behar SM Furlong ST Brenner MB. Recognition of a lipid antigen by CD1-restricted alpha beta+ T cells. Nature (1994) 372(6507):691–4. doi: 10.1038/372691a0 7527500

[52] Lee CH Chen LC Yu CC Lin WH Lin VC Huang CY. Prognostic value of CD1B in localised prostate cancer. Int J Environ Res Public Health (2019) 16(23):4723. doi: 10.3390/ijerph16234723 31783478 PMC6926967

[53] Chen W Huang J Xiong J Fu P Chen C Liu Y. Identification of a tumor microenvironment-related gene signature indicative of disease prognosis and treatment response in colon cancer. Oxid Med Cell Longevity (2021) 2021:6290261. doi: 10.1155/2021/6290261 PMC8420973 34497681

[54] Li N Li Y Zheng P Zhan X. Cancer stemness-based prognostic immune-related gene signatures in lung adenocarcinoma and lung squamous cell carcinoma. Front Endocrinol (2021) 12:755805. doi: 10.3389/fendo.2021.755805 PMC8567176 34745015

[55] Yuan J Yuan B Zeng L Liu B Chen Y Meng X. Identification and validation of tumor microenvironment-related genes of prognostic value in lung adenocarcinoma. Oncol Lett (2020) 20(2):1772–80. doi: 10.3892/ol.2020.11735 PMC7377199 32724420

[56] Zhang H Qin C Gan H Guo X Zhang L. Construction of an immunogenomic risk score for prognostication in colon cancer. Front Genet (2020) 11:499. doi: 10.3389/fgene.2020.00499 32508884 PMC7253627

[57] Lee BC Avraham S Imamoto A Avraham HK. Identification of the nonreceptor tyrosine kinase MATK/CHK as an essential regulator of immune cells using Matk/CHK-deficient mice. Blood (2006) 108(3):904–7. doi: 10.1182/blood-2005-12-4885 PMC1895851 16574955

[58] Chüeh AC Advani G Foroutan M Smith J Ng N Nandurkar H. CSK-homologous kinase (CHK/MATK) is a potential colorectal cancer tumour suppressor gene epigenetically silenced by promoter methylation. Oncogene (2021) 40(17):3015–29. doi: 10.1038/s41388-021-01755-z 33767439

[59] Park JH Jonas SF Bataillon G Criscitiello C Salgado R Loi S. Prognostic value of tumor-infiltrating lymphocytes in patients with early-stage triple-negative breast cancers (TNBC) who did not receive adjuvant chemotherapy. Ann Oncol Off J Eur Soc Med Oncol (2019) 30(12):1941–9. doi: 10.1093/annonc/mdz395 31566659

[60] Tomioka N Azuma M Ikarashi M Yamamoto M Sato M Watanabe KI. The therapeutic candidate for immune checkpoint inhibitors elucidated by the status of tumor-infiltrating lymphocytes (TILs) and programmed death ligand 1 (PD-L1) expression in triple negative breast cancer (TNBC). Breast Cancer (Tokyo Japan) (2018) 25(1):34–42. doi: 10.1007/s12282-017-0781-0 28488168

[61] Gao ZH Li CX Liu M Jiang JY. Predictive and prognostic role of tumour-infiltrating lymphocytes in breast cancer patients with different molecular subtypes: a meta-analysis. BMC Cancer (2020) 20(1):1150. doi: 10.1186/s12885-020-07654-y 33238978 PMC7690150

[62] Zimmerli D Brambillasca CS Talens F Bhin J Linstra R Romanens L. MYC promotes immune-suppression in triple-negative breast cancer *via* inhibition of interferon signaling. Nat Commun (2022) 13(1):6579. doi: 10.1038/s41467-022-34000-6 36323660 PMC9630413

[63] Gao C Li H Liu C Xu X Zhuang J Zhou C. Tumor mutation burden and immune invasion characteristics in triple negative breast cancer: Genome high-throughput data analysis. Front Immunol (2021) 12:650491. doi: 10.3389/fimmu.2021.650491 33968045 PMC8097167

[64] Cheng J Ding X Xu S Zhu B Jia Q. Gene expression profiling identified TP53(Mut)PIK3CA(Wild) as a potential biomarker for patients with triple-negative breast cancer treated with immune checkpoint inhibitors. Oncol Lett (2020) 19(4):2817–24. doi: 10.3892/ol.2020.11381 PMC7068237 32218835

[65] Zhang X Li J Yang Q Wang Y Li X Liu Y. Tumor mutation burden and JARID2 gene alteration are associated with short disease-free survival in locally advanced triple-negative breast cancer. Ann Trans Med (2020) 8(17):1052. doi: 10.21037/atm-20-3773 PMC7576007 33145271

