# Supplementary figures and images for: Immune checkpoints expression patterns in early-stage triple-negative breast cancer predict prognosis and remodel the tumor immune microenvironment
Source: Front Immunol. 2023 Feb 6;14:1073550. doi: 10.3389/fimmu.2023.1073550 (PMC9939840; doi:10.3389/fimmu.2023.1073550)

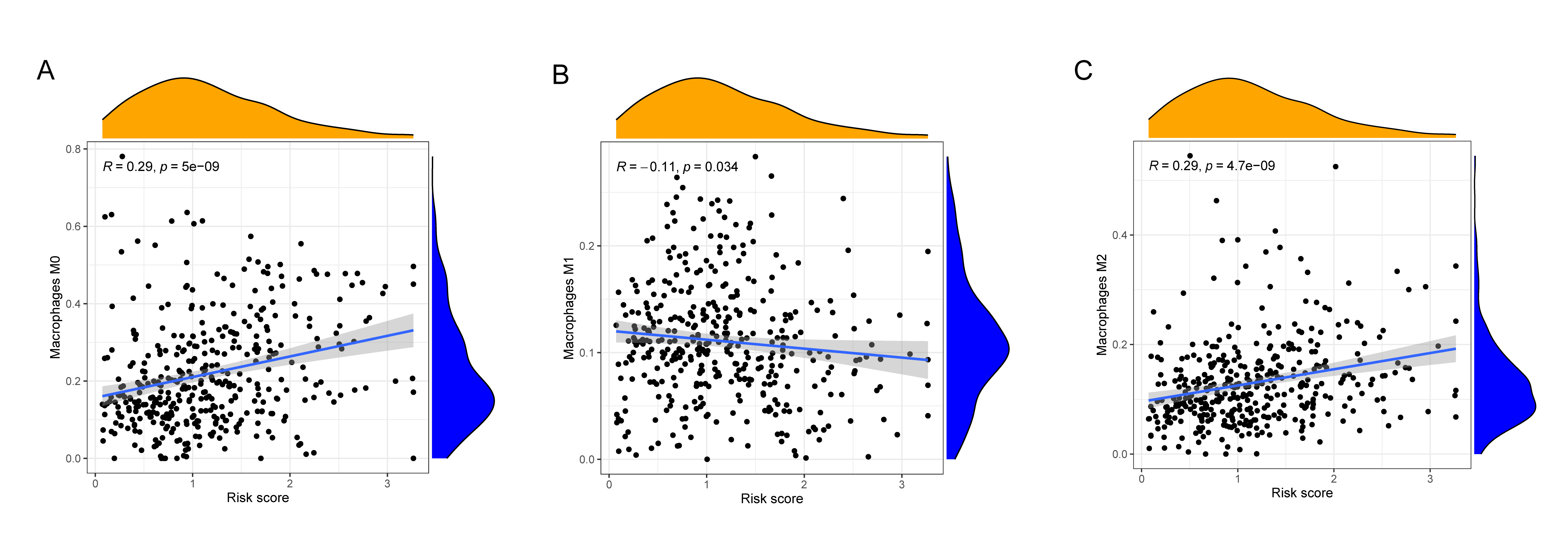

Supplement: Supplementary Figure 1 — Functional enrichment for ICG-related genes using GO annotation analysis. [file Image_1.jpeg]

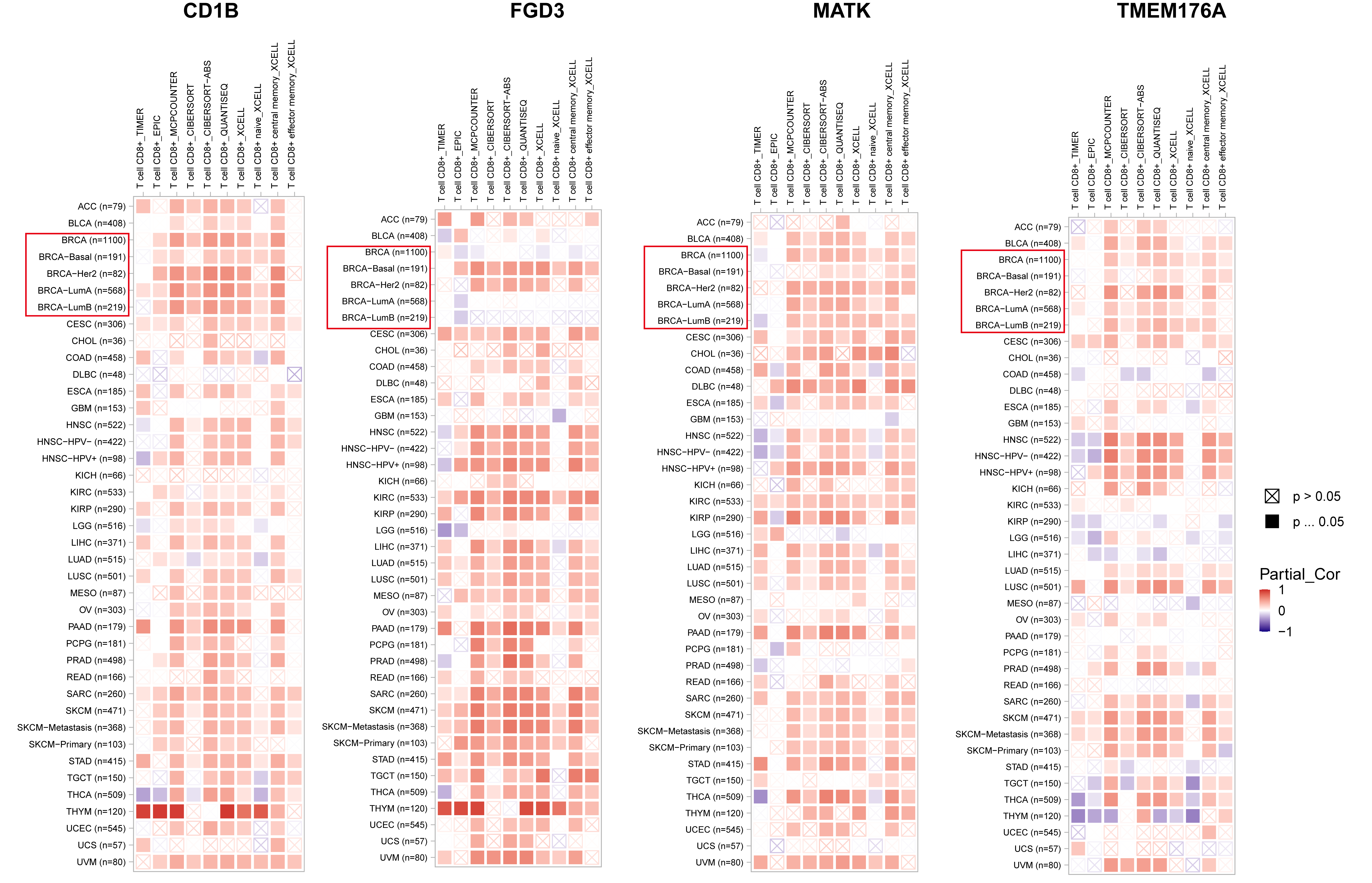

Supplement: Supplementary Figure 2 — Functional enrichment for ICG-related genes using KEGG annotation analysis. [file Image_2.tif]

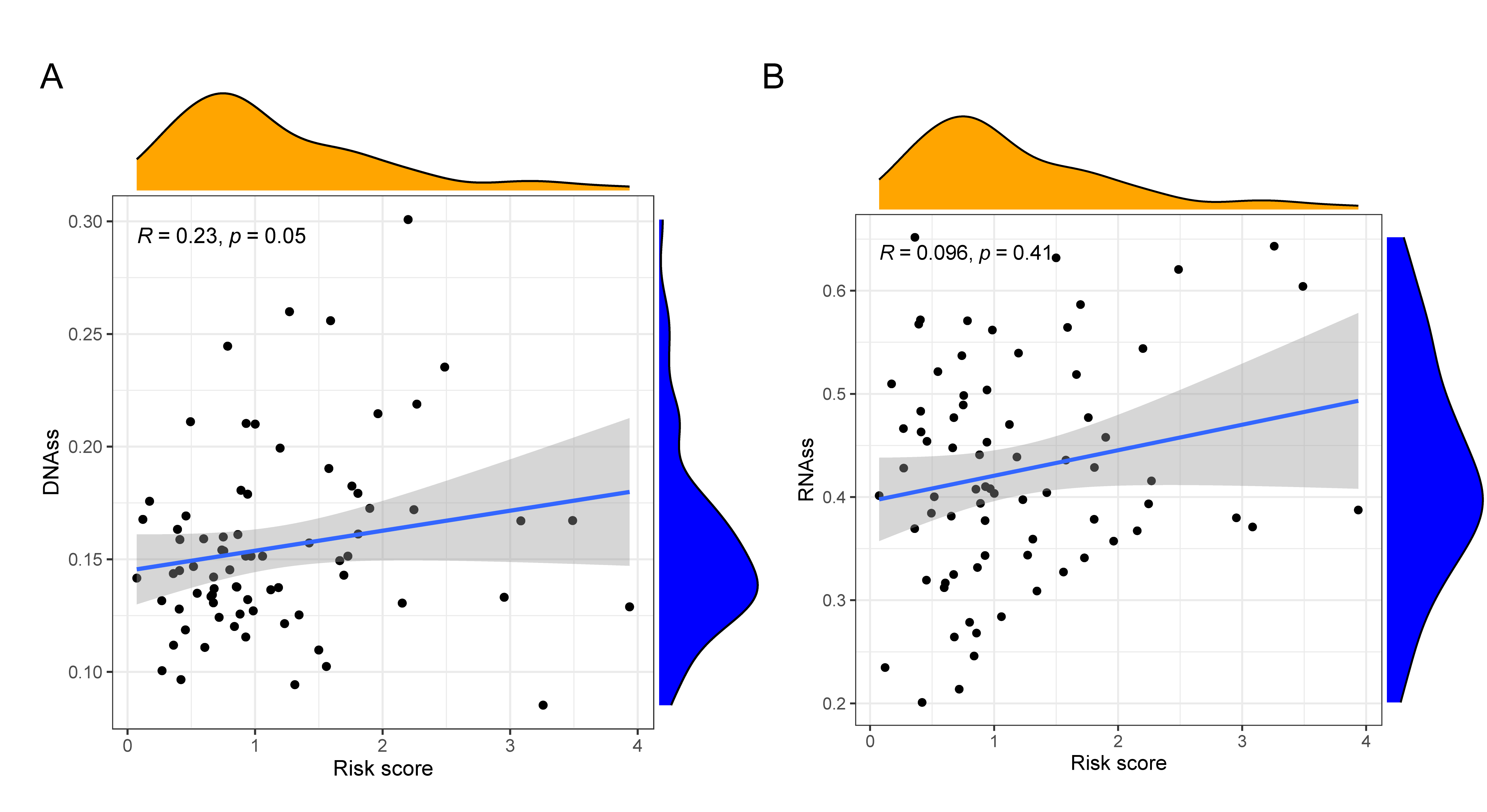

Supplement: Supplementary Figure 3 — Multivariable Cox-regression analysis adjusting for stage, IRS and the infiltration of immune cells. [file Image_3.jpeg]

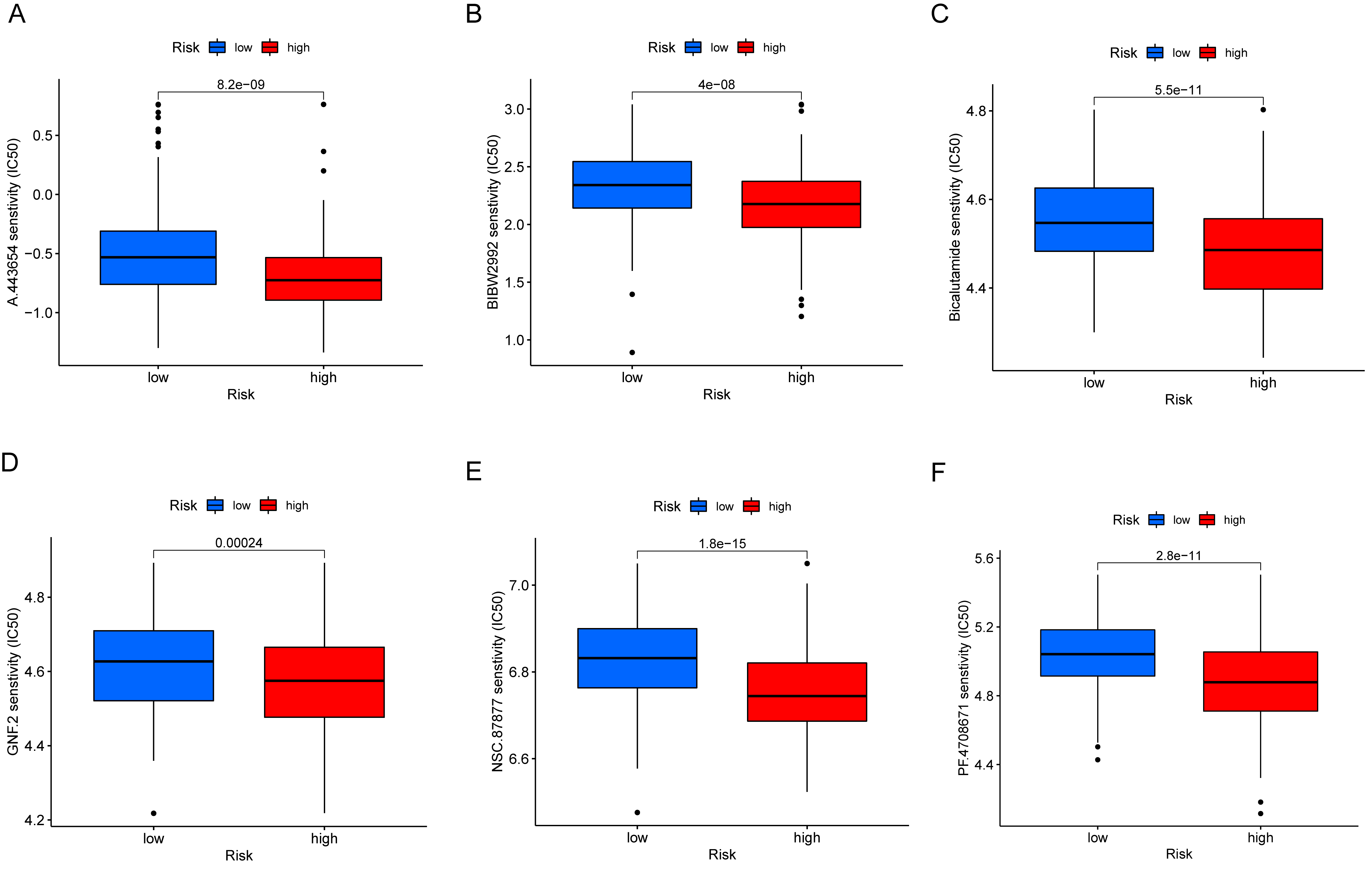

Supplement: Supplementary Figure 4 — Correlation analysis between ICGs-related riskscore and the level of M0 macrophages, M1 macrophages and M2 macrophages. [file Image_4.tif]

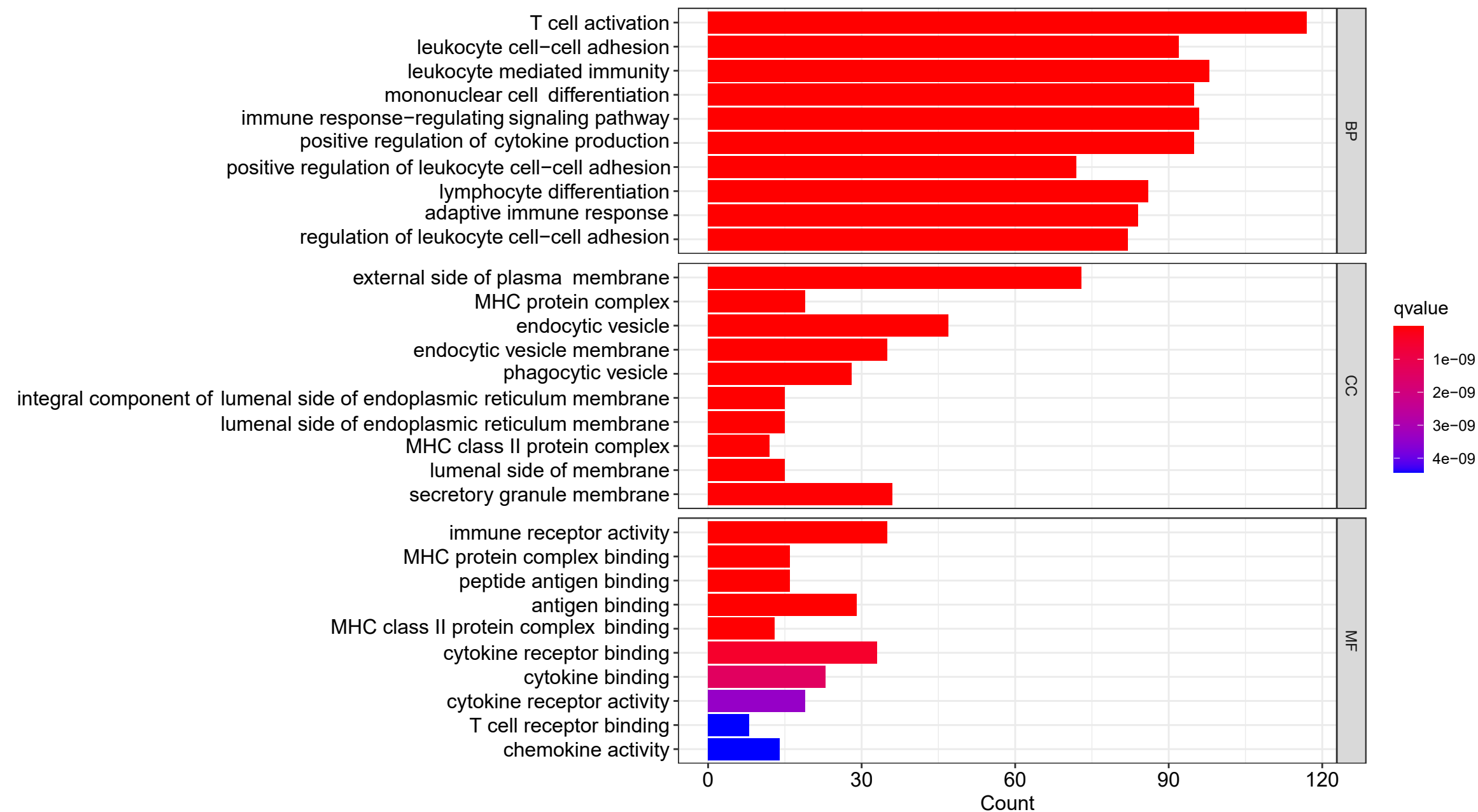

Supplement: Supplementary Figure 5 — The relationship between CD8 T cells infiltration and IRS model genes by other immune-cell infiltration algorithms. [file DataSheet_1.pdf]

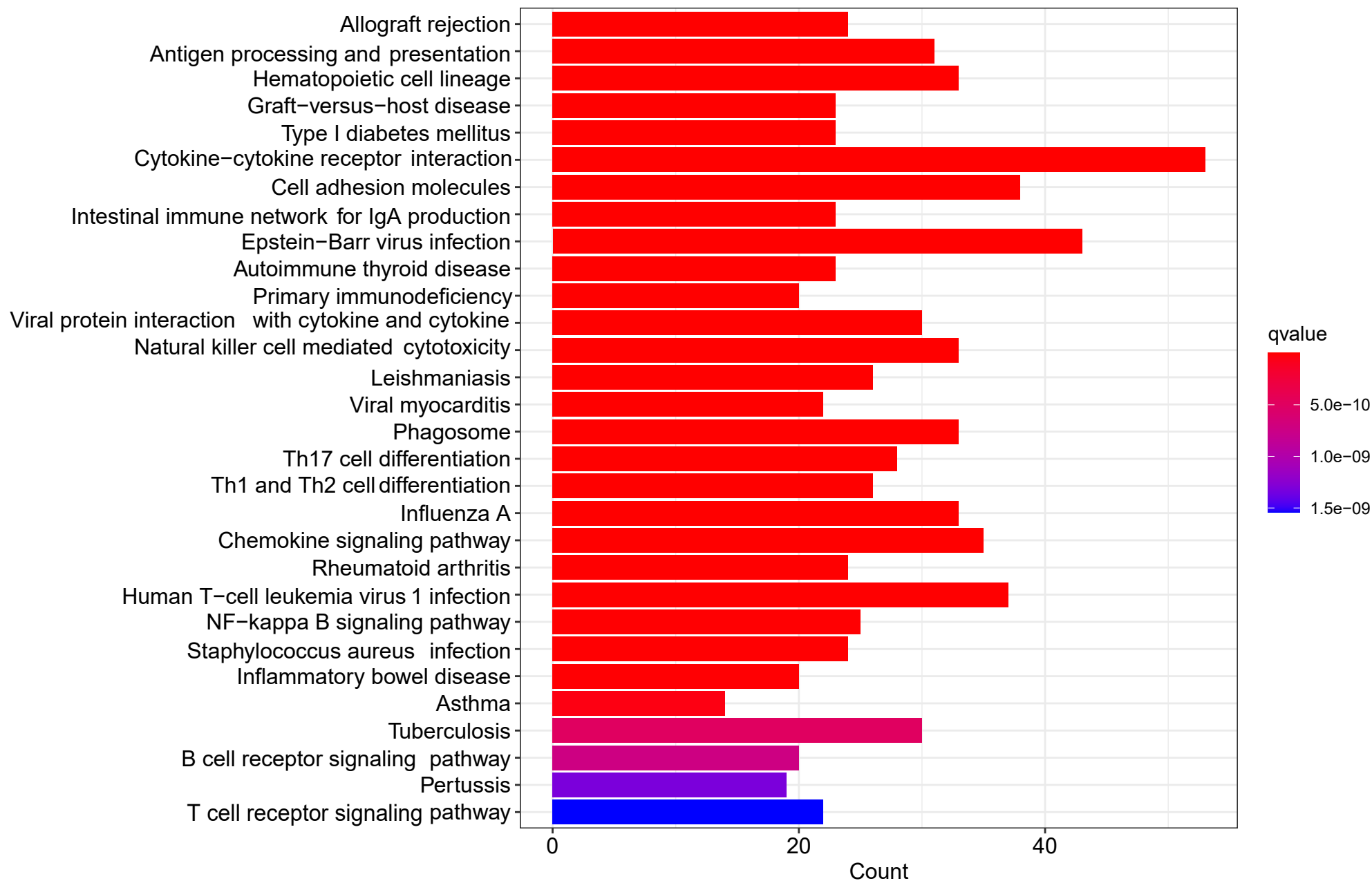

Supplement: Supplementary Figure 6 — Correlation analysis between IRS and the stemness index DNAss and RNAss. [file DataSheet_2.pdf]

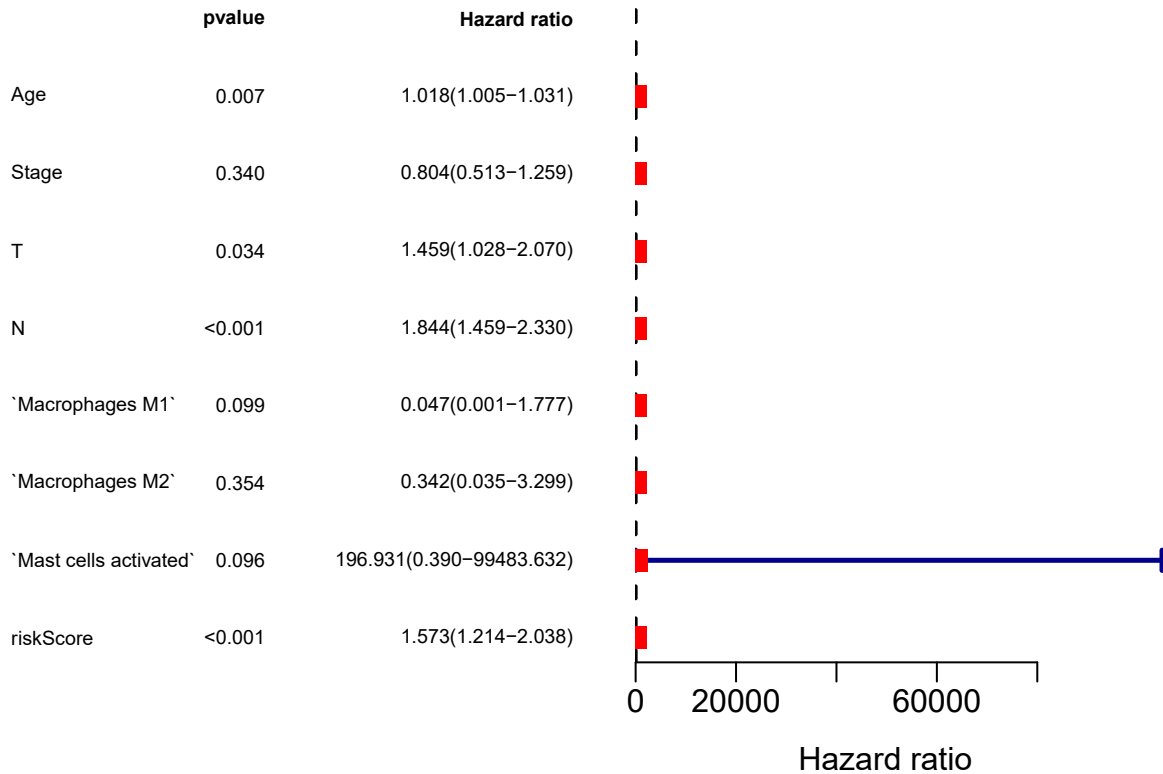

Supplement: Supplementary Figure 7 — Drug sensitivity analysis in different IRS groups. [file DataSheet_3.pdf]
