# Supplementary material for: Immune checkpoints expression patterns in early-stage triple-negative breast cancer predict prognosis and remodel the tumor immune microenvironment
Source: Front Immunol. 2023 Feb 6;14:1073550. doi: 10.3389/fimmu.2023.1073550 (PMC9939840; doi:10.3389/fimmu.2023.1073550)
Supplement: Supplementary file 9 [file Table_2.docx]

| Table. S2 Univariate Cox regression analysis and Kaplan–Meier (KM) log-rank test of ICGs in TNBC. | | | | | |
| --- | --- | --- | --- | --- | --- |
| id | HR | HR.95L | HR.95H | pvalue | km |
| BTN2A2 | 0.579007 | 0.41186 | 0.813988 | 0.001666 | 0.000669 |
| BTNL3 | 0.848769 | 0.290851 | 2.4769 | 0.764121 | 0.336481 |
| CEACAM1 | 0.885851 | 0.793455 | 0.989008 | 0.031034 | 0.001025 |
| IDO1 | 0.845237 | 0.768624 | 0.929485 | 0.000524 | 6.13E-05 |
| TDO2 | 0.944417 | 0.807257 | 1.104883 | 0.475073 | 0.06331 |
| VTCN1 | 0.971591 | 0.891937 | 1.058359 | 0.509031 | 0.059752 |
| ADORA2A | 0.722636 | 0.540091 | 0.96688 | 0.028766 | 0.00857 |
| BTN3A1 | 0.755751 | 0.62173 | 0.918661 | 0.004927 | 0.002651 |
| CD276 | 1.053091 | 0.842203 | 1.316786 | 0.650036 | 0.002935 |
| CD274 | 0.724188 | 0.553247 | 0.947947 | 0.018819 | 0.002521 |
| PDCD1LG2 | 0.746222 | 0.572817 | 0.97212 | 0.030043 | 0.001454 |
| PDCD1 | 0.805726 | 0.661079 | 0.982023 | 0.032383 | 0.001006 |
| CD28 | 0.833053 | 0.632749 | 1.096767 | 0.193016 | 0.025328 |
| CD80 | 0.741743 | 0.528315 | 1.041391 | 0.084403 | 0.00018 |
| CD86 | 0.902764 | 0.76213 | 1.069349 | 0.236437 | 0.02665 |
| CTLA4 | 0.733048 | 0.615367 | 0.873233 | 0.000505 | 5.57E-05 |
| ICOS | 0.758566 | 0.640875 | 0.89787 | 0.001317 | 9.47E-05 |
| ICOSLG | 1.174897 | 0.871336 | 1.584214 | 0.29057 | 0.056136 |
| CD160 | 0.639923 | 0.409617 | 0.999719 | 0.049856 | 0.020225 |
| BTLA | 0.728874 | 0.565525 | 0.939405 | 0.014574 | 4.99E-05 |
| TNFRSF14 | 0.767534 | 0.635761 | 0.926619 | 0.005905 | 0.000506 |
| TNFSF14 | 0.764842 | 0.579505 | 1.009454 | 0.058291 | 0.011512 |
| TNFSF9 | 1.054178 | 0.822457 | 1.351183 | 0.676968 | 0.006596 |
| TNFRSF9 | 0.857085 | 0.666003 | 1.102991 | 0.230804 | 0.043488 |
| TNFSF4 | 1.022211 | 0.824275 | 1.267678 | 0.841435 | 0.05503 |
| TNFRSF4 | 0.802307 | 0.646492 | 0.995676 | 0.045576 | 0.003958 |
| CD70 | 0.886217 | 0.706751 | 1.111255 | 0.295444 | 0.148886 |
| CD27 | 0.825653 | 0.713909 | 0.954887 | 0.009819 | 0.000158 |
| CD40 | 0.778917 | 0.641747 | 0.945407 | 0.011472 | 0.00131 |
| CD40LG | 0.833654 | 0.648572 | 1.071552 | 0.155485 | 0.001454 |
| LGALS9 | 0.943636 | 0.791563 | 1.124926 | 0.517603 | 0.129882 |
| TNFSF18 | 1.370982 | 0.931451 | 2.01792 | 0.109622 | 0.055729 |
| TNFRSF18 | 0.994608 | 0.827305 | 1.195743 | 0.954116 | 0.126209 |
| CD47 | 0.844059 | 0.667824 | 1.066802 | 0.15596 | 0.019003 |
| SIRPA | 0.9177 | 0.770247 | 1.093381 | 0.336546 | 0.021747 |
| CD226 | 0.541518 | 0.35222 | 0.832552 | 0.005189 | 0.001193 |
| TIGIT | 0.858459 | 0.687563 | 1.071832 | 0.177823 | 0.006221 |
| PVR | 1.145057 | 0.922312 | 1.421596 | 0.21973 | 0.028016 |
| BTN2A1 | 0.741472 | 0.496238 | 1.107896 | 0.144323 | 0.000374 |
| HLA-DOB | 0.784193 | 0.673837 | 0.912621 | 0.00168 | 0.000294 |
| HLA-G | 0.8207 | 0.705118 | 0.955227 | 0.010728 | 0.000507 |
| KIR2DL1 | 0.958436 | 0.466299 | 1.969979 | 0.908059 | 0.033769 |
| KIR2DL4 | 0.706318 | 0.528039 | 0.944789 | 0.019149 | 0.001896 |
| HLA-A | 0.907413 | 0.84691 | 0.972239 | 0.005786 | 0.001708 |
| HLA-B | 0.820318 | 0.714936 | 0.941233 | 0.004753 | 0.000422 |
| HLA-C | 0.869598 | 0.749817 | 1.008513 | 0.064624 | 0.02617 |
| HLA-DMA | 0.830248 | 0.719829 | 0.957604 | 0.010621 | 0.00012 |
| HLA-DMB | 0.830632 | 0.722643 | 0.954758 | 0.009015 | 0.000821 |
| HLA-DOA | 0.843122 | 0.739622 | 0.961106 | 0.010661 | 0.001076 |
| HLA-DPA1 | 0.840037 | 0.747207 | 0.944399 | 0.003529 | 0.000329 |
| HLA-DPB1 | 0.842458 | 0.721242 | 0.984045 | 0.03055 | 0.003352 |
| HLA-DQA1 | 0.869346 | 0.784589 | 0.963259 | 0.007469 | 0.001441 |
| HLA-DQB1 | 0.977689 | 0.880469 | 1.085643 | 0.672848 | 0.120487 |
| HLA-DRA | 0.841892 | 0.749449 | 0.945737 | 0.003731 | 0.000304 |
| HLA-DRB1 | 0.95784 | 0.896295 | 1.023612 | 0.203654 | 0.001401 |
| HLA-E | 0.830965 | 0.696811 | 0.990946 | 0.039283 | 0.012301 |
| HLA-F | 0.800901 | 0.698084 | 0.918861 | 0.00154 | 0.001234 |
| LAG3 | 0.899704 | 0.792505 | 1.021404 | 0.102514 | 0.04629 |
| KIR2DS4 | 0.735517 | 0.457089 | 1.183543 | 0.205636 | 0.032799 |
| KIR3DL1 | 0.3424 | 0.169978 | 0.689725 | 0.002704 | 0.000163 |
| KIR3DL3 | 0.321094 | 0.12538 | 0.822314 | 0.017899 | 4.01E-07 |
| IDO2 | 0.797769 | 0.516427 | 1.232382 | 0.308553 | 0.007246 |

HR:Hazard ratios; HR.95L: 95% lower confidence intervals; HR.95H: 95% higher confidence intervals;
