# Supplementary material for: Immune checkpoints expression patterns in early-stage triple-negative breast cancer predict prognosis and remodel the tumor immune microenvironment
Source: Front Immunol. 2023 Feb 6;14:1073550. doi: 10.3389/fimmu.2023.1073550 (PMC9939840; doi:10.3389/fimmu.2023.1073550)
Supplement: Supplementary file 10 [file Table_3.docx]

| Table. S3 Identification of DEGs in two ICGs clusters using “limma” algorithm. | | | | | | |
| --- | --- | --- | --- | --- | --- | --- |
| id | logFC | AveExpr | t | P.Value | adj.P.Val | B |
| APOBEC3G | 1.449543 | 6.104606 | 22.97367 | 2.64E-76 | 4.26E-72 | 163.1382 |
| GBP4 | 1.893236 | 7.211367 | 22.22497 | 5.61E-73 | 4.52E-69 | 155.5309 |
| HLA-DMA | 1.422954 | 9.891566 | 21.61187 | 3.03E-70 | 1.63E-66 | 149.2843 |
| IRF1 | 1.481114 | 8.1215 | 21.50648 | 8.93E-70 | 3.45E-66 | 148.2093 |
| HLA-E | 1.204747 | 9.477903 | 21.48895 | 1.07E-69 | 3.45E-66 | 148.0304 |
| HLA-DMB | 1.447604 | 9.274698 | 21.17999 | 2.56E-68 | 6.88E-65 | 144.8774 |
| GZMA | 1.804977 | 6.714286 | 20.37779 | 9.79E-65 | 2.26E-61 | 136.6841 |
| CD2 | 1.85125 | 7.391581 | 20.27335 | 2.87E-64 | 5.78E-61 | 135.6171 |
| IL18BP | 1.272598 | 7.301607 | 20.19525 | 6.40E-64 | 1.15E-60 | 134.8193 |
| CXCR3 | 1.368255 | 5.44425 | 20.12088 | 1.38E-63 | 2.22E-60 | 134.0596 |
| CCL5 | 2.001689 | 9.534034 | 20.02149 | 3.82E-63 | 5.61E-60 | 133.0443 |
| PSMB10 | 1.078218 | 9.132757 | 19.97464 | 6.19E-63 | 8.32E-60 | 132.5658 |
| HLA-B | 1.436091 | 11.84726 | 19.94103 | 8.75E-63 | 1.09E-59 | 132.2225 |
| GBP5 | 1.967781 | 6.661522 | 19.91479 | 1.15E-62 | 1.32E-59 | 131.9545 |
| CD3D | 1.873653 | 7.324075 | 19.89778 | 1.36E-62 | 1.47E-59 | 131.7808 |
| PSMB9 | 1.607273 | 7.561426 | 19.74879 | 6.31E-62 | 6.36E-59 | 130.2593 |
| WAS | 1.284701 | 7.58057 | 19.65299 | 1.69E-61 | 1.60E-58 | 129.2812 |
| CD53 | 1.250133 | 6.814943 | 19.63885 | 1.95E-61 | 1.75E-58 | 129.1368 |
| HLA-DRA | 1.580422 | 12.00554 | 19.52875 | 6.06E-61 | 5.14E-58 | 128.0129 |
| NKG7 | 1.874008 | 7.093873 | 19.46097 | 1.22E-60 | 9.81E-58 | 127.3212 |
| HLA-DQA1 | 1.864342 | 9.234822 | 19.40306 | 2.20E-60 | 1.69E-57 | 126.7303 |
| HLA-DPA1 | 1.587574 | 10.32826 | 19.3069 | 5.92E-60 | 4.34E-57 | 125.7493 |
| HLA-F | 1.470557 | 7.718698 | 19.27478 | 8.23E-60 | 5.77E-57 | 125.4217 |
| HCST | 1.486661 | 8.321563 | 19.25219 | 1.04E-59 | 6.72E-57 | 125.1912 |
| ARHGAP9 | 1.317376 | 6.488639 | 19.25185 | 1.04E-59 | 6.72E-57 | 125.1877 |
| IDO1 | 2.185789 | 6.412679 | 19.17207 | 2.36E-59 | 1.47E-56 | 124.3741 |
| PLEK | 1.472974 | 7.501694 | 19.16675 | 2.50E-59 | 1.49E-56 | 124.3199 |
| BTN3A3 | 1.045831 | 6.174877 | 19.14555 | 3.10E-59 | 1.79E-56 | 124.1038 |
| CXCR6 | 1.10952 | 5.13839 | 19.11103 | 4.42E-59 | 2.46E-56 | 123.7518 |
| ICOS | 1.218268 | 5.436045 | 19.05648 | 7.74E-59 | 4.16E-56 | 123.1957 |
| GIMAP4 | 1.338507 | 8.338928 | 18.96789 | 1.92E-58 | 9.99E-56 | 122.2929 |
| CD8A | 1.660591 | 6.550848 | 18.95621 | 2.16E-58 | 1.09E-55 | 122.1739 |
| PRF1 | 1.355842 | 5.620541 | 18.94272 | 2.49E-58 | 1.22E-55 | 122.0365 |
| IL21R | 1.099888 | 5.236108 | 18.87111 | 5.18E-58 | 2.46E-55 | 121.307 |
| CD247 | 1.552123 | 6.556262 | 18.82161 | 8.61E-58 | 3.97E-55 | 120.8028 |
| HLA-DOA | 1.502588 | 7.166765 | 18.77855 | 1.34E-57 | 6.00E-55 | 120.3645 |
| ITGB7 | 1.180141 | 5.885653 | 18.74514 | 1.88E-57 | 8.22E-55 | 120.0243 |
| CYBB | 1.444932 | 6.966468 | 18.66709 | 4.19E-57 | 1.78E-54 | 119.2299 |
| CASP1 | 1.131276 | 7.208838 | 18.6311 | 6.06E-57 | 2.51E-54 | 118.8637 |
| PTPN7 | 1.166507 | 5.500212 | 18.61044 | 7.49E-57 | 3.02E-54 | 118.6535 |
| HCLS1 | 1.346636 | 8.084962 | 18.56153 | 1.24E-56 | 4.86E-54 | 118.156 |
| CST7 | 1.312072 | 6.219975 | 18.55317 | 1.35E-56 | 5.17E-54 | 118.071 |
| RAC2 | 1.540969 | 8.319705 | 18.53774 | 1.58E-56 | 5.92E-54 | 117.914 |
| NCF4 | 1.03459 | 6.072249 | 18.46047 | 3.48E-56 | 1.28E-53 | 117.1284 |
| SLAMF1 | 1.044554 | 4.912028 | 18.44105 | 4.24E-56 | 1.52E-53 | 116.931 |
| EVI2B | 1.432824 | 7.019006 | 18.42567 | 4.97E-56 | 1.73E-53 | 116.7747 |
| FGL2 | 1.4578 | 7.10836 | 18.42417 | 5.04E-56 | 1.73E-53 | 116.7594 |
| IRF8 | 1.528982 | 7.253367 | 18.38369 | 7.63E-56 | 2.56E-53 | 116.348 |
| GZMB | 1.913408 | 6.578749 | 18.3621 | 9.52E-56 | 3.13E-53 | 116.1287 |
| FERMT3 | 1.171392 | 7.351624 | 18.34016 | 1.19E-55 | 3.84E-53 | 115.9058 |
| CD86 | 1.133643 | 6.948401 | 18.26044 | 2.69E-55 | 8.51E-53 | 115.0961 |
| BTN3A1 | 1.028659 | 6.595056 | 18.19288 | 5.37E-55 | 1.67E-52 | 114.4103 |
| IL10RA | 1.203022 | 6.265556 | 18.16601 | 7.06E-55 | 2.15E-52 | 114.1376 |
| CORO1A | 1.315697 | 7.194066 | 18.09723 | 1.43E-54 | 4.26E-52 | 113.4398 |
| CD4 | 1.041646 | 6.354394 | 18.0871 | 1.58E-54 | 4.64E-52 | 113.3371 |
| SASH3 | 1.248656 | 6.419996 | 17.96335 | 5.59E-54 | 1.61E-51 | 112.0825 |
| CD48 | 1.507603 | 6.539177 | 17.95161 | 6.30E-54 | 1.78E-51 | 111.9635 |
| C1QA | 1.319303 | 7.812035 | 17.93104 | 7.78E-54 | 2.16E-51 | 111.755 |
| PYHIN1 | 1.043979 | 5.130098 | 17.90847 | 9.79E-54 | 2.68E-51 | 111.5264 |
| TNFRSF1B | 1.016126 | 6.483184 | 17.87499 | 1.38E-53 | 3.69E-51 | 111.1873 |
| ARHGAP25 | 1.120479 | 6.212603 | 17.87375 | 1.39E-53 | 3.69E-51 | 111.1747 |
| SAMSN1 | 1.033362 | 5.724262 | 17.73877 | 5.52E-53 | 1.44E-50 | 109.8085 |
| CD7 | 1.355472 | 5.881471 | 17.63596 | 1.57E-52 | 4.03E-50 | 108.7687 |
| MAP4K1 | 1.282459 | 6.269455 | 17.62728 | 1.72E-52 | 4.33E-50 | 108.681 |
| CXCL9 | 2.471238 | 8.643431 | 17.60776 | 2.09E-52 | 5.20E-50 | 108.4837 |
| NCKAP1L | 1.0349 | 5.834106 | 17.58117 | 2.75E-52 | 6.71E-50 | 108.215 |
| RASAL3 | 1.295568 | 6.646789 | 17.56646 | 3.19E-52 | 7.68E-50 | 108.0664 |
| LTA | 1.050304 | 5.177019 | 17.51194 | 5.55E-52 | 1.32E-49 | 107.5158 |
| IFNG | 1.114642 | 4.664056 | 17.48677 | 7.17E-52 | 1.68E-49 | 107.2617 |
| C1QB | 1.46823 | 10.0664 | 17.47877 | 7.78E-52 | 1.79E-49 | 107.1809 |
| CYTIP | 1.053156 | 5.709153 | 17.43367 | 1.23E-51 | 2.79E-49 | 106.7256 |
| ITGB2 | 1.313117 | 9.418804 | 17.42244 | 1.38E-51 | 3.09E-49 | 106.6123 |
| PSTPIP1 | 0.927091 | 5.119857 | 17.42071 | 1.40E-51 | 3.10E-49 | 106.5949 |
| CD74 | 1.34782 | 8.985175 | 17.39542 | 1.81E-51 | 3.95E-49 | 106.3397 |
| MYO1G | 0.934951 | 5.560517 | 17.38698 | 1.98E-51 | 4.25E-49 | 106.2546 |
| CD38 | 1.47043 | 5.474597 | 17.36891 | 2.37E-51 | 5.04E-49 | 106.0724 |
| GPSM3 | 1.075682 | 6.699915 | 17.29678 | 4.94E-51 | 1.03E-48 | 105.3451 |
| DOCK2 | 1.22177 | 6.905364 | 17.292 | 5.18E-51 | 1.07E-48 | 105.297 |
| ITK | 1.204421 | 5.439156 | 17.28126 | 5.78E-51 | 1.18E-48 | 105.1888 |
| CD3G | 1.138554 | 5.186202 | 17.21872 | 1.09E-50 | 2.20E-48 | 104.5587 |
| DOCK10 | 1.043795 | 6.45577 | 17.18743 | 1.50E-50 | 2.98E-48 | 104.2437 |
| LAPTM5 | 1.152843 | 8.801546 | 17.13637 | 2.51E-50 | 4.94E-48 | 103.7298 |
| STX11 | 0.998362 | 5.764279 | 17.10558 | 3.43E-50 | 6.67E-48 | 103.42 |
| SLC7A7 | 1.130293 | 7.387474 | 17.08909 | 4.05E-50 | 7.78E-48 | 103.2541 |
| ABI3 | 0.799687 | 5.874482 | 17.06917 | 4.96E-50 | 9.35E-48 | 103.0538 |
| LAG3 | 1.464512 | 6.006477 | 17.06861 | 4.99E-50 | 9.35E-48 | 103.0482 |
| TAP1 | 1.537898 | 9.858931 | 17.05348 | 5.81E-50 | 1.08E-47 | 102.896 |
| SLA | 0.938935 | 5.840575 | 17.00773 | 9.23E-50 | 1.69E-47 | 102.4362 |
| GBP1 | 1.547143 | 8.111534 | 16.99025 | 1.10E-49 | 2.00E-47 | 102.2606 |
| NCF1 | 0.98557 | 5.274189 | 16.98033 | 1.22E-49 | 2.18E-47 | 102.161 |
| PRKCB | 1.394038 | 5.906523 | 16.96786 | 1.38E-49 | 2.45E-47 | 102.0357 |
| ZBED2 | 0.888164 | 4.568135 | 16.96485 | 1.42E-49 | 2.50E-47 | 102.0054 |
| LPXN | 1.090447 | 8.058932 | 16.96151 | 1.47E-49 | 2.56E-47 | 101.9719 |
| IL18RAP | 0.761204 | 4.688951 | 16.93275 | 1.97E-49 | 3.38E-47 | 101.683 |
| GZMK | 1.8195 | 6.512405 | 16.88485 | 3.20E-49 | 5.43E-47 | 101.2023 |
| GNLY | 1.732563 | 6.407106 | 16.84725 | 4.68E-49 | 7.86E-47 | 100.825 |
| ARHGAP30 | 0.999554 | 6.327608 | 16.82207 | 6.03E-49 | 1.00E-46 | 100.5725 |
| AIM2 | 1.368024 | 5.69741 | 16.80325 | 7.29E-49 | 1.20E-46 | 100.3837 |
| ADAMDEC1 | 1.771801 | 6.614057 | 16.75324 | 1.21E-48 | 1.97E-46 | 99.88251 |
| CTLA4 | 1.087039 | 5.415063 | 16.7386 | 1.40E-48 | 2.26E-46 | 99.73583 |
| STAT4 | 1.086463 | 5.605433 | 16.73532 | 1.45E-48 | 2.31E-46 | 99.70296 |
| FASLG | 0.871542 | 4.585877 | 16.69977 | 2.07E-48 | 3.28E-46 | 99.34682 |
| ZNF683 | 1.23458 | 5.384613 | 16.57531 | 7.26E-48 | 1.14E-45 | 98.10148 |
| NLRC5 | 0.988005 | 5.59996 | 16.55596 | 8.82E-48 | 1.37E-45 | 97.90801 |
| LILRB4 | 1.164386 | 6.160245 | 16.55382 | 9.01E-48 | 1.38E-45 | 97.88655 |
| GMFG | 1.108546 | 7.293388 | 16.51664 | 1.31E-47 | 1.99E-45 | 97.51503 |
| EPSTI1 | 1.350195 | 7.604093 | 16.50482 | 1.48E-47 | 2.23E-45 | 97.39692 |
| CXCL13 | 2.088232 | 6.681837 | 16.41823 | 3.52E-47 | 5.27E-45 | 96.53232 |
| DOCK8 | 1.021882 | 6.018141 | 16.40976 | 3.84E-47 | 5.68E-45 | 96.44781 |
| TBC1D10C | 1.293559 | 5.871726 | 16.39931 | 4.26E-47 | 6.25E-45 | 96.34355 |
| SLA2 | 0.916896 | 4.850653 | 16.39773 | 4.33E-47 | 6.29E-45 | 96.32784 |
| C2 | 1.058464 | 6.388889 | 16.34556 | 7.31E-47 | 1.05E-44 | 95.80753 |
| SAMD3 | 0.758696 | 4.669352 | 16.34503 | 7.35E-47 | 1.05E-44 | 95.80227 |
| GZMH | 1.294004 | 5.668874 | 16.33655 | 8.00E-47 | 1.13E-44 | 95.71773 |
| CTSW | 1.149444 | 5.324121 | 16.31792 | 9.65E-47 | 1.35E-44 | 95.53204 |
| SP140 | 0.941772 | 4.951223 | 16.30131 | 1.14E-46 | 1.59E-44 | 95.36658 |
| SPI1 | 0.966305 | 6.624204 | 16.22899 | 2.35E-46 | 3.25E-44 | 94.64636 |
| SH2D1A | 1.109054 | 5.312901 | 16.22265 | 2.51E-46 | 3.43E-44 | 94.58321 |
| PDCD1 | 0.960596 | 4.769581 | 16.21371 | 2.74E-46 | 3.72E-44 | 94.49425 |
| FGD2 | 1.004079 | 6.239269 | 16.20184 | 3.09E-46 | 4.15E-44 | 94.37621 |
| CD6 | 1.277094 | 6.356505 | 16.179 | 3.89E-46 | 5.18E-44 | 94.14893 |
| ACAP1 | 1.122281 | 5.636377 | 16.14929 | 5.23E-46 | 6.92E-44 | 93.85355 |
| APOBEC3F | 0.935284 | 5.598757 | 16.14726 | 5.34E-46 | 7.00E-44 | 93.83338 |
| GIMAP5 | 1.033815 | 5.733498 | 16.14165 | 5.65E-46 | 7.35E-44 | 93.77757 |
| CD27 | 1.329336 | 5.907341 | 16.13581 | 5.99E-46 | 7.73E-44 | 93.7195 |
| PSMB8 | 1.19991 | 7.521159 | 16.10497 | 8.15E-46 | 1.04E-43 | 93.41305 |
| UBD | 2.491335 | 7.978806 | 16.07089 | 1.15E-45 | 1.46E-43 | 93.07451 |
| IL2RG | 1.040396 | 5.589507 | 16.05836 | 1.30E-45 | 1.64E-43 | 92.95003 |
| APBB1IP | 1.038349 | 6.852735 | 15.97918 | 2.87E-45 | 3.56E-43 | 92.16432 |
| SLC15A3 | 1.117323 | 8.232319 | 15.9791 | 2.87E-45 | 3.56E-43 | 92.16352 |
| VAV1 | 0.846192 | 5.287596 | 15.97711 | 2.93E-45 | 3.60E-43 | 92.14382 |
| IGSF6 | 1.086112 | 6.2494 | 15.96149 | 3.42E-45 | 4.18E-43 | 91.9889 |
| STAT1 | 1.430387 | 9.97019 | 15.91494 | 5.44E-45 | 6.60E-43 | 91.52758 |
| HLA-DPB1 | 1.208228 | 7.445481 | 15.90101 | 6.25E-45 | 7.53E-43 | 91.38961 |
| CSF2RA | 0.990793 | 5.848198 | 15.85366 | 1.00E-44 | 1.19E-42 | 90.9207 |
| TNFRSF9 | 0.713085 | 4.743956 | 15.83501 | 1.21E-44 | 1.42E-42 | 90.73616 |
| GIMAP6 | 1.069464 | 6.581437 | 15.80381 | 1.65E-44 | 1.93E-42 | 90.42752 |
| LCP2 | 0.720232 | 5.274348 | 15.79356 | 1.83E-44 | 2.12E-42 | 90.32608 |
| BTN3A2 | 1.028176 | 6.833411 | 15.77035 | 2.30E-44 | 2.65E-42 | 90.09662 |
| GBP2 | 1.259658 | 9.133705 | 15.76487 | 2.43E-44 | 2.78E-42 | 90.04245 |
| CD40 | 0.949216 | 6.043263 | 15.70987 | 4.20E-44 | 4.77E-42 | 89.49906 |
| TLR8 | 1.015076 | 5.126825 | 15.65016 | 7.60E-44 | 8.57E-42 | 88.90975 |
| ARHGAP15 | 1.055398 | 6.228174 | 15.63062 | 9.23E-44 | 1.03E-41 | 88.71691 |
| KLHL6 | 0.873428 | 5.091585 | 15.61976 | 1.03E-43 | 1.14E-41 | 88.60985 |
| C1QC | 1.249973 | 9.713833 | 15.61866 | 1.04E-43 | 1.15E-41 | 88.59898 |
| HLA-DOB | 1.235058 | 6.082517 | 15.61491 | 1.08E-43 | 1.18E-41 | 88.56207 |
| APOBEC3H | 0.76428 | 4.757021 | 15.58515 | 1.45E-43 | 1.58E-41 | 88.26872 |
| MYO1F | 0.782116 | 5.37642 | 15.54973 | 2.06E-43 | 2.23E-41 | 87.9197 |
| TYROBP | 1.145683 | 9.648592 | 15.50491 | 3.21E-43 | 3.45E-41 | 87.47849 |
| C1orf162 | 1.00102 | 7.924856 | 15.50221 | 3.30E-43 | 3.52E-41 | 87.45187 |
| PARVG | 0.95201 | 6.123129 | 15.4966 | 3.49E-43 | 3.70E-41 | 87.39667 |
| PSME2 | 0.864926 | 9.184654 | 15.42836 | 6.86E-43 | 7.23E-41 | 86.72565 |
| HLA-H | 1.478697 | 8.071026 | 15.40698 | 8.47E-43 | 8.88E-41 | 86.51552 |
| ZAP70 | 1.028934 | 5.002525 | 15.38041 | 1.10E-42 | 1.15E-40 | 86.25451 |
| BTK | 0.784 | 5.152074 | 15.33213 | 1.78E-42 | 1.84E-40 | 85.78065 |
| CD37 | 1.279504 | 6.331809 | 15.31963 | 2.01E-42 | 2.06E-40 | 85.65801 |
| LY96 | 0.989898 | 8.062261 | 15.30755 | 2.26E-42 | 2.31E-40 | 85.53959 |
| CD52 | 1.739142 | 8.169223 | 15.22792 | 4.97E-42 | 5.04E-40 | 84.7592 |
| LY9 | 0.780675 | 4.517249 | 15.20147 | 6.45E-42 | 6.50E-40 | 84.50032 |
| IGFLR1 | 0.876382 | 6.022846 | 15.17724 | 8.19E-42 | 8.20E-40 | 84.26323 |
| CARD16 | 0.705743 | 5.239644 | 15.16477 | 9.26E-42 | 9.22E-40 | 84.14117 |
| CD5 | 1.013905 | 5.194634 | 15.1376 | 1.21E-41 | 1.20E-39 | 83.87551 |
| INPP5D | 0.928013 | 5.844752 | 15.13177 | 1.28E-41 | 1.26E-39 | 83.81854 |
| CD3E | 1.16846 | 5.631031 | 15.12294 | 1.40E-41 | 1.37E-39 | 83.73224 |
| P2RY10 | 1.0291 | 5.0079 | 15.07738 | 2.19E-41 | 2.13E-39 | 83.28722 |
| SLAMF7 | 1.034554 | 5.193372 | 15.07123 | 2.32E-41 | 2.25E-39 | 83.22716 |
| TNFRSF14 | 0.941421 | 8.194785 | 15.0637 | 2.50E-41 | 2.40E-39 | 83.15363 |
| PTPRC | 1.086185 | 5.540962 | 15.04714 | 2.95E-41 | 2.81E-39 | 82.99208 |
| GIMAP7 | 1.320407 | 7.292529 | 15.02532 | 3.65E-41 | 3.46E-39 | 82.77916 |
| P2RY8 | 1.115215 | 5.568283 | 15.01165 | 4.18E-41 | 3.94E-39 | 82.64585 |
| AKNA | 0.98928 | 6.556914 | 15.00814 | 4.32E-41 | 4.05E-39 | 82.6116 |
| SRGN | 1.39533 | 9.174749 | 14.97346 | 6.07E-41 | 5.67E-39 | 82.27361 |
| S1PR4 | 0.88246 | 4.953078 | 14.96206 | 6.79E-41 | 6.30E-39 | 82.16248 |
| TNFSF13B | 1.361448 | 7.396348 | 14.94017 | 8.42E-41 | 7.76E-39 | 81.94927 |
| IL12RB1 | 0.660117 | 4.56521 | 14.93314 | 9.02E-41 | 8.27E-39 | 81.8809 |
| PIM2 | 1.284061 | 7.413692 | 14.92911 | 9.39E-41 | 8.56E-39 | 81.84168 |
| LST1 | 0.973697 | 6.379163 | 14.89513 | 1.31E-40 | 1.19E-38 | 81.51093 |
| ALOX5 | 1.080435 | 7.733171 | 14.76929 | 4.49E-40 | 4.04E-38 | 80.28838 |
| CCL4 | 1.192039 | 6.647081 | 14.75872 | 4.98E-40 | 4.46E-38 | 80.18579 |
| TRAT1 | 0.885325 | 4.747236 | 14.73214 | 6.45E-40 | 5.75E-38 | 79.92807 |
| TRIM21 | 0.747409 | 6.847377 | 14.73064 | 6.55E-40 | 5.80E-38 | 79.9135 |
| TMEM140 | 0.812146 | 7.12848 | 14.70121 | 8.73E-40 | 7.69E-38 | 79.62825 |
| IL7R | 1.481362 | 6.761427 | 14.70018 | 8.81E-40 | 7.73E-38 | 79.61831 |
| FCER1G | 1.126466 | 9.053505 | 14.68948 | 9.78E-40 | 8.53E-38 | 79.51465 |
| PARP14 | 1.042866 | 7.293568 | 14.65249 | 1.40E-39 | 1.22E-37 | 79.15646 |
| RNASE6 | 1.032155 | 6.728201 | 14.63205 | 1.71E-39 | 1.48E-37 | 78.95868 |
| TIGIT | 0.761419 | 4.709335 | 14.6072 | 2.18E-39 | 1.87E-37 | 78.71838 |
| KLRB1 | 1.194566 | 5.827617 | 14.54827 | 3.87E-39 | 3.31E-37 | 78.14899 |
| UBASH3A | 0.764778 | 4.664721 | 14.48267 | 7.33E-39 | 6.22E-37 | 77.51601 |
| CSF1R | 1.076425 | 8.720035 | 14.47024 | 8.27E-39 | 6.98E-37 | 77.39615 |
| LAT2 | 0.801435 | 5.974357 | 14.46038 | 9.10E-39 | 7.65E-37 | 77.30116 |
| DOK2 | 0.805687 | 5.236725 | 14.43679 | 1.14E-38 | 9.56E-37 | 77.07386 |
| RASSF5 | 1.035504 | 6.848153 | 14.41756 | 1.38E-38 | 1.15E-36 | 76.88876 |
| LAP3 | 0.950584 | 8.757919 | 14.41395 | 1.43E-38 | 1.18E-36 | 76.85395 |
| CCR2 | 0.874544 | 4.870402 | 14.38191 | 1.95E-38 | 1.60E-36 | 76.54562 |
| WIPF1 | 0.93816 | 6.639311 | 14.36414 | 2.31E-38 | 1.90E-36 | 76.37474 |
| ST8SIA4 | 0.607517 | 5.168304 | 14.35763 | 2.46E-38 | 2.01E-36 | 76.31215 |
| EOMES | 1.084432 | 5.431726 | 14.35725 | 2.47E-38 | 2.01E-36 | 76.30848 |
| UBE2L6 | 1.067801 | 7.645396 | 14.3521 | 2.60E-38 | 2.10E-36 | 76.25903 |
| PSME1 | 0.776412 | 10.38072 | 14.29015 | 4.74E-38 | 3.80E-36 | 75.66393 |
| B2M | 0.961388 | 12.14251 | 14.2468 | 7.20E-38 | 5.75E-36 | 75.24815 |
| CD69 | 1.151707 | 5.696716 | 14.21773 | 9.53E-38 | 7.58E-36 | 74.96949 |
| CLEC4A | 0.83613 | 5.591705 | 14.1823 | 1.34E-37 | 1.06E-35 | 74.63015 |
| CXCL10 | 1.949932 | 9.396538 | 14.17008 | 1.51E-37 | 1.19E-35 | 74.51318 |
| TNFAIP3 | 0.902518 | 7.069971 | 14.16497 | 1.59E-37 | 1.24E-35 | 74.46428 |
| CD84 | 0.936812 | 5.696154 | 14.1423 | 1.97E-37 | 1.54E-35 | 74.24742 |
| TRPV2 | 0.847421 | 6.221714 | 14.1324 | 2.17E-37 | 1.68E-35 | 74.15279 |
| CLECL1 | 0.779654 | 4.840639 | 14.10167 | 2.92E-37 | 2.25E-35 | 73.85904 |
| SEMA4D | 0.822683 | 7.272903 | 14.03082 | 5.77E-37 | 4.43E-35 | 73.1828 |
| LCK | 0.799998 | 5.218204 | 14.01936 | 6.44E-37 | 4.92E-35 | 73.07359 |
| EVI2A | 0.97462 | 6.170128 | 13.97903 | 9.49E-37 | 7.22E-35 | 72.68922 |
| JAK2 | 0.659747 | 5.615677 | 13.96008 | 1.14E-36 | 8.62E-35 | 72.50883 |
| BATF2 | 1.114683 | 5.289447 | 13.94878 | 1.27E-36 | 9.56E-35 | 72.4013 |
| PTPN6 | 0.823082 | 7.547054 | 13.91224 | 1.80E-36 | 1.35E-34 | 72.05373 |
| CD72 | 0.751462 | 5.419813 | 13.80356 | 5.09E-36 | 3.79E-34 | 71.02194 |
| HLA-G | 1.142827 | 6.025654 | 13.77981 | 6.39E-36 | 4.73E-34 | 70.79692 |
| THEMIS | 0.662986 | 4.452845 | 13.74233 | 9.13E-36 | 6.73E-34 | 70.44207 |
| AIF1 | 1.009513 | 7.377906 | 13.73837 | 9.49E-36 | 6.96E-34 | 70.4046 |
| RGS18 | 0.67167 | 4.869355 | 13.72483 | 1.08E-35 | 7.88E-34 | 70.27651 |
| MS4A6A | 1.164754 | 7.541047 | 13.72439 | 1.08E-35 | 7.88E-34 | 70.27232 |
| ACSL5 | 1.028618 | 6.989401 | 13.70242 | 1.34E-35 | 9.67E-34 | 70.06458 |
| FGR | 0.921232 | 6.501057 | 13.66513 | 1.91E-35 | 1.37E-33 | 69.71221 |
| PTPN22 | 0.696862 | 4.868858 | 13.59596 | 3.68E-35 | 2.64E-33 | 69.05968 |
| P2RY13 | 0.698957 | 4.802233 | 13.59387 | 3.75E-35 | 2.68E-33 | 69.03993 |
| IFI35 | 1.047561 | 7.732598 | 13.56916 | 4.75E-35 | 3.37E-33 | 68.80717 |
| LTB | 1.640114 | 7.406162 | 13.53507 | 6.56E-35 | 4.64E-33 | 68.48629 |
| CMKLR1 | 0.705998 | 5.17554 | 13.50036 | 9.11E-35 | 6.42E-33 | 68.15988 |
| SELL | 1.373835 | 5.98039 | 13.43168 | 1.75E-34 | 1.22E-32 | 67.51495 |
| PLCB2 | 0.751375 | 5.737138 | 13.42854 | 1.80E-34 | 1.25E-32 | 67.4855 |
| KLRD1 | 0.640908 | 4.791244 | 13.42581 | 1.85E-34 | 1.28E-32 | 67.4599 |
| ARHGAP4 | 0.862993 | 6.614723 | 13.42038 | 1.94E-34 | 1.34E-32 | 67.40897 |
| LCP1 | 1.211093 | 9.790701 | 13.37621 | 2.95E-34 | 2.02E-32 | 66.99506 |
| TBXAS1 | 0.809607 | 6.103339 | 13.35589 | 3.57E-34 | 2.44E-32 | 66.8049 |
| VCAM1 | 1.303459 | 8.090506 | 13.3128 | 5.36E-34 | 3.65E-32 | 66.40186 |
| IFI30 | 0.815849 | 6.481894 | 13.31033 | 5.49E-34 | 3.72E-32 | 66.3788 |
| LGALS9 | 0.938709 | 6.727888 | 13.30439 | 5.80E-34 | 3.92E-32 | 66.32322 |
| BTLA | 0.694255 | 4.550662 | 13.25083 | 9.61E-34 | 6.46E-32 | 65.82322 |
| PRDM1 | 0.836351 | 6.583918 | 13.2466 | 1.00E-33 | 6.69E-32 | 65.78376 |
| NAPSB | 1.25954 | 6.498259 | 13.23804 | 1.08E-33 | 7.22E-32 | 65.70385 |
| GIMAP2 | 0.796902 | 5.314412 | 13.21234 | 1.38E-33 | 9.08E-32 | 65.46436 |
| CCR7 | 1.348708 | 5.798477 | 13.15764 | 2.31E-33 | 1.51E-31 | 64.95507 |
| LYZ | 1.820151 | 9.062513 | 13.13704 | 2.80E-33 | 1.83E-31 | 64.76347 |
| XAF1 | 1.143347 | 7.097757 | 13.11328 | 3.49E-33 | 2.26E-31 | 64.5427 |
| IL15RA | 0.750668 | 5.399396 | 13.04434 | 6.66E-33 | 4.30E-31 | 63.90306 |
| IFI16 | 1.010621 | 7.779875 | 13.01982 | 8.37E-33 | 5.38E-31 | 63.67584 |
| PLAC8 | 1.145969 | 5.364399 | 12.98801 | 1.13E-32 | 7.21E-31 | 63.3814 |
| PARP9 | 0.8766 | 7.752202 | 12.96095 | 1.45E-32 | 9.24E-31 | 63.13123 |
| TRIM69 | 0.585963 | 5.379747 | 12.9445 | 1.69E-32 | 1.07E-30 | 62.97921 |
| NLRP3 | 0.62085 | 5.086805 | 12.94181 | 1.73E-32 | 1.10E-30 | 62.95434 |
| RAB8B | 0.74391 | 6.915483 | 12.90314 | 2.48E-32 | 1.56E-30 | 62.59739 |
| SLCO2B1 | 1.018769 | 7.439429 | 12.89088 | 2.78E-32 | 1.73E-30 | 62.48426 |
| IL23A | 0.666121 | 5.077387 | 12.87634 | 3.19E-32 | 1.98E-30 | 62.35021 |
| CTSC | 1.017249 | 8.115776 | 12.87437 | 3.25E-32 | 2.01E-30 | 62.33211 |
| MNDA | 0.907785 | 5.80796 | 12.86726 | 3.47E-32 | 2.13E-30 | 62.26655 |
| PPM1M | 0.587693 | 6.866493 | 12.83658 | 4.61E-32 | 2.83E-30 | 61.98401 |
| CD79A | 1.85218 | 6.764626 | 12.82277 | 5.24E-32 | 3.20E-30 | 61.85687 |
| PAG1 | 0.738316 | 5.624257 | 12.80194 | 6.36E-32 | 3.87E-30 | 61.6653 |
| SLC9A9 | 0.693479 | 5.456946 | 12.68476 | 1.88E-31 | 1.14E-29 | 60.59015 |
| FPR3 | 1.007592 | 7.225765 | 12.67506 | 2.06E-31 | 1.24E-29 | 60.50138 |
| SNX20 | 0.638123 | 4.729815 | 12.6606 | 2.35E-31 | 1.41E-29 | 60.36901 |
| CD79B | 1.305611 | 6.602219 | 12.65003 | 2.59E-31 | 1.55E-29 | 60.27236 |
| SOCS1 | 0.765472 | 5.588633 | 12.62861 | 3.16E-31 | 1.89E-29 | 60.07648 |
| PIK3CD | 0.806976 | 5.876008 | 12.62707 | 3.20E-31 | 1.91E-29 | 60.06245 |
| SEL1L3 | 1.013458 | 7.025401 | 12.60975 | 3.75E-31 | 2.23E-29 | 59.90422 |
| MEI1 | 0.60175 | 4.613767 | 12.60886 | 3.79E-31 | 2.24E-29 | 59.89606 |
| ITGAX | 0.860645 | 6.350478 | 12.53465 | 7.49E-31 | 4.40E-29 | 59.21923 |
| VAMP5 | 0.843535 | 8.402559 | 12.50876 | 9.50E-31 | 5.55E-29 | 58.98357 |
| PLEKHO2 | 0.667769 | 6.736985 | 12.49566 | 1.07E-30 | 6.24E-29 | 58.86437 |
| TRAF3IP3 | 0.958066 | 5.576142 | 12.49453 | 1.08E-30 | 6.28E-29 | 58.85411 |
| LAMP3 | 1.332226 | 6.837505 | 12.48925 | 1.14E-30 | 6.57E-29 | 58.80607 |
| EMP3 | 0.798736 | 8.540728 | 12.48142 | 1.22E-30 | 7.04E-29 | 58.73488 |
| ZNF831 | 0.614668 | 4.41858 | 12.46024 | 1.48E-30 | 8.51E-29 | 58.54242 |
| SYK | 0.844278 | 7.269881 | 12.43314 | 1.90E-30 | 1.09E-28 | 58.29644 |
| TAGAP | 0.753236 | 5.122934 | 12.42925 | 1.97E-30 | 1.12E-28 | 58.2611 |
| TNFRSF4 | 0.734114 | 6.300351 | 12.40765 | 2.40E-30 | 1.36E-28 | 58.06525 |
| TNFRSF17 | 1.303085 | 5.371743 | 12.40599 | 2.44E-30 | 1.38E-28 | 58.05017 |
| TMEM176B | 0.818988 | 6.666719 | 12.36768 | 3.46E-30 | 1.95E-28 | 57.70312 |
| ICAM3 | 0.857457 | 6.543227 | 12.36751 | 3.46E-30 | 1.95E-28 | 57.70164 |
| SLFN11 | 0.853845 | 7.188206 | 12.36644 | 3.50E-30 | 1.96E-28 | 57.69192 |
| DEF6 | 0.668541 | 6.531854 | 12.34861 | 4.11E-30 | 2.29E-28 | 57.53061 |
| TRAF1 | 0.652613 | 5.46593 | 12.33952 | 4.47E-30 | 2.48E-28 | 57.44834 |
| GPR65 | 0.72406 | 5.372801 | 12.33636 | 4.60E-30 | 2.54E-28 | 57.41983 |
| IGHG1 | 2.585031 | 9.369227 | 12.33251 | 4.77E-30 | 2.62E-28 | 57.38499 |
| FCGR1A | 0.607424 | 5.248022 | 12.28043 | 7.66E-30 | 4.19E-28 | 56.91466 |
| POU2AF1 | 1.115293 | 5.238956 | 12.227 | 1.25E-29 | 6.79E-28 | 56.43313 |
| TRANK1 | 0.766615 | 6.124239 | 12.2231 | 1.29E-29 | 7.01E-28 | 56.39803 |
| PLEKHA2 | 0.7082 | 7.189075 | 12.20924 | 1.46E-29 | 7.92E-28 | 56.27324 |
| LILRB3 | 0.686671 | 5.664975 | 12.20781 | 1.48E-29 | 8.00E-28 | 56.26035 |
| TAPBP | 0.744612 | 9.65262 | 12.19689 | 1.64E-29 | 8.77E-28 | 56.16219 |
| PTAFR | 0.705944 | 5.366138 | 12.1886 | 1.76E-29 | 9.42E-28 | 56.08765 |
| NFS1 | 0.870756 | 6.831047 | 12.18562 | 1.81E-29 | 9.65E-28 | 56.06083 |
| MCOLN2 | 0.855663 | 6.237639 | 12.17637 | 1.97E-29 | 1.05E-27 | 55.97774 |
| CLEC12A | 0.593094 | 4.483215 | 12.1662 | 2.16E-29 | 1.14E-27 | 55.88629 |
| LYL1 | 0.753687 | 6.556438 | 12.1482 | 2.54E-29 | 1.34E-27 | 55.72467 |
| PLCL2 | 0.809602 | 6.191321 | 12.11845 | 3.33E-29 | 1.75E-27 | 55.45786 |
| IRF9 | 0.716029 | 7.900738 | 12.09834 | 3.99E-29 | 2.09E-27 | 55.27759 |
| LRMP | 0.918623 | 5.203595 | 12.07763 | 4.82E-29 | 2.51E-27 | 55.09214 |
| GPR183 | 1.05155 | 6.914968 | 12.07103 | 5.11E-29 | 2.65E-27 | 55.03309 |
| LAIR1 | 0.673114 | 5.469862 | 12.0188 | 8.19E-29 | 4.23E-27 | 54.56625 |
| SP110 | 0.718011 | 6.613254 | 12.00875 | 8.97E-29 | 4.62E-27 | 54.47647 |
| IL15 | 0.700215 | 5.130022 | 12.00854 | 8.98E-29 | 4.62E-27 | 54.47459 |
| NPL | 0.738993 | 6.511874 | 11.99669 | 9.99E-29 | 5.12E-27 | 54.36887 |
| C1S | 1.098892 | 9.463261 | 11.95038 | 1.52E-28 | 7.71E-27 | 53.95605 |
| TAPBPL | 0.717661 | 6.346551 | 11.94923 | 1.53E-28 | 7.77E-27 | 53.94589 |
| HLA-C | 1.010722 | 8.376699 | 11.92932 | 1.83E-28 | 9.26E-27 | 53.76862 |
| STK10 | 0.602689 | 5.851649 | 11.90598 | 2.26E-28 | 1.14E-26 | 53.56111 |
| DOCK11 | 0.809359 | 6.216443 | 11.86653 | 3.22E-28 | 1.61E-26 | 53.21077 |
| GCH1 | 0.642136 | 5.724085 | 11.86204 | 3.35E-28 | 1.67E-26 | 53.17093 |
| IGKC | 1.811875 | 9.214812 | 11.85749 | 3.49E-28 | 1.74E-26 | 53.13055 |
| SPOCK2 | 1.307633 | 7.596689 | 11.84594 | 3.87E-28 | 1.91E-26 | 53.02811 |
| RTP4 | 1.097933 | 6.261613 | 11.80513 | 5.57E-28 | 2.75E-26 | 52.66662 |
| ISG20 | 0.981539 | 7.848019 | 11.80217 | 5.72E-28 | 2.81E-26 | 52.6404 |
| C1orf54 | 0.637681 | 7.565684 | 11.80021 | 5.82E-28 | 2.85E-26 | 52.62303 |
| CD14 | 0.968097 | 8.912236 | 11.7969 | 5.99E-28 | 2.93E-26 | 52.59378 |
| STK17B | 0.681354 | 6.218544 | 11.76723 | 7.81E-28 | 3.81E-26 | 52.33139 |
| PTGER4 | 0.761676 | 6.15642 | 11.74955 | 9.14E-28 | 4.44E-26 | 52.17527 |
| RASSF4 | 0.843229 | 6.299884 | 11.73142 | 1.07E-27 | 5.21E-26 | 52.01518 |
| PVRIG | 0.782946 | 4.868944 | 11.72651 | 1.12E-27 | 5.42E-26 | 51.97187 |
| FCN1 | 1.035281 | 5.728711 | 11.71884 | 1.20E-27 | 5.79E-26 | 51.90422 |
| APOC1 | 1.129731 | 9.23546 | 11.69522 | 1.48E-27 | 7.10E-26 | 51.69604 |
| APOBEC3C | 0.681956 | 5.825644 | 11.68312 | 1.65E-27 | 7.88E-26 | 51.58951 |
| CYSLTR1 | 0.621187 | 4.958083 | 11.67181 | 1.83E-27 | 8.67E-26 | 51.48992 |
| ITGA4 | 0.610153 | 4.989736 | 11.66371 | 1.96E-27 | 9.28E-26 | 51.4187 |
| IL32 | 1.098508 | 7.575414 | 11.65375 | 2.14E-27 | 1.01E-25 | 51.33103 |
| CD163 | 1.195088 | 7.744033 | 11.64884 | 2.24E-27 | 1.05E-25 | 51.28788 |
| CD274 | 0.607349 | 4.672326 | 11.6407 | 2.41E-27 | 1.13E-25 | 51.21636 |
| SKAP1 | 0.991534 | 6.139326 | 11.59524 | 3.60E-27 | 1.67E-25 | 50.81725 |
| DENND2D | 0.765911 | 7.282975 | 11.58388 | 3.98E-27 | 1.85E-25 | 50.71765 |
| SERPING1 | 0.846699 | 8.945081 | 11.57866 | 4.17E-27 | 1.93E-25 | 50.67184 |
| CD19 | 1.238112 | 5.157201 | 11.55486 | 5.15E-27 | 2.37E-25 | 50.46336 |
| TLR7 | 0.871081 | 5.737676 | 11.53815 | 5.97E-27 | 2.73E-25 | 50.31717 |
| SERPINB1 | 0.66967 | 7.51277 | 11.52982 | 6.42E-27 | 2.92E-25 | 50.24431 |
| TAP2 | 0.674354 | 5.561996 | 11.50098 | 8.28E-27 | 3.75E-25 | 49.99228 |
| ALOX5AP | 1.082649 | 8.378449 | 11.49563 | 8.68E-27 | 3.92E-25 | 49.94553 |
| CD40LG | 0.650307 | 4.503397 | 11.48393 | 9.63E-27 | 4.34E-25 | 49.84342 |
| MZB1 | 1.667611 | 7.034141 | 11.45431 | 1.25E-26 | 5.61E-25 | 49.58503 |
| STAMBPL1 | 0.679775 | 5.788329 | 11.44531 | 1.35E-26 | 6.06E-25 | 49.50666 |
| TNFAIP8 | 0.664368 | 5.62347 | 11.43322 | 1.50E-26 | 6.72E-25 | 49.40131 |
| NCR3 | 0.609455 | 4.41106 | 11.42555 | 1.61E-26 | 7.17E-25 | 49.33457 |
| BATF | 0.949664 | 6.388557 | 11.41327 | 1.79E-26 | 7.97E-25 | 49.22769 |
| MPP1 | 0.61571 | 6.163329 | 11.40645 | 1.90E-26 | 8.43E-25 | 49.16841 |
| FGD3 | 0.831617 | 6.634766 | 11.40391 | 1.95E-26 | 8.60E-25 | 49.14636 |
| CD300A | 0.718026 | 6.010177 | 11.40135 | 1.99E-26 | 8.77E-25 | 49.12408 |
| ADAP2 | 0.711717 | 6.992184 | 11.37374 | 2.54E-26 | 1.11E-24 | 48.88414 |
| EFHD2 | 0.632891 | 8.384968 | 11.33123 | 3.68E-26 | 1.61E-24 | 48.51546 |
| IFFO1 | 0.681282 | 6.45016 | 11.31762 | 4.14E-26 | 1.81E-24 | 48.39752 |
| GPR18 | 0.756024 | 4.740553 | 11.30914 | 4.46E-26 | 1.95E-24 | 48.32407 |
| MPEG1 | 0.737664 | 5.611488 | 11.30664 | 4.56E-26 | 1.98E-24 | 48.30243 |
| PPP1R16B | 0.944621 | 5.596925 | 11.28458 | 5.53E-26 | 2.40E-24 | 48.11161 |
| CYBA | 0.810993 | 9.589094 | 11.26071 | 6.81E-26 | 2.95E-24 | 47.9053 |
| CELF2 | 0.742586 | 5.429798 | 11.25342 | 7.26E-26 | 3.12E-24 | 47.84236 |
| IL18R1 | 0.611938 | 5.045861 | 11.2314 | 8.80E-26 | 3.77E-24 | 47.65235 |
| DDX60 | 0.867804 | 6.145211 | 11.21675 | 9.99E-26 | 4.28E-24 | 47.526 |
| IL16 | 0.623118 | 5.228636 | 11.18344 | 1.33E-25 | 5.70E-24 | 47.23921 |
| DPEP2 | 0.622692 | 4.990727 | 11.17316 | 1.46E-25 | 6.21E-24 | 47.15074 |
| DRAM1 | 0.731814 | 7.288058 | 11.15861 | 1.66E-25 | 7.01E-24 | 47.02562 |
| TMC8 | 0.595055 | 4.8018 | 11.14725 | 1.83E-25 | 7.72E-24 | 46.92799 |
| RFTN1 | 0.7775 | 8.031481 | 11.1168 | 2.38E-25 | 1.00E-23 | 46.66663 |
| CCR1 | 0.718172 | 5.675564 | 11.11551 | 2.41E-25 | 1.01E-23 | 46.65557 |
| PTK2B | 0.611603 | 5.58623 | 11.11224 | 2.48E-25 | 1.04E-23 | 46.62756 |
| CLEC10A | 0.726036 | 4.682475 | 11.10301 | 2.68E-25 | 1.12E-23 | 46.5484 |
| CDC42SE2 | 0.592642 | 7.111018 | 11.09713 | 2.82E-25 | 1.18E-23 | 46.49804 |
| PNOC | 0.689156 | 4.49649 | 11.09017 | 3.00E-25 | 1.25E-23 | 46.43841 |
| LY86 | 0.77626 | 6.885871 | 11.01423 | 5.77E-25 | 2.38E-23 | 45.78911 |
| APOE | 0.943077 | 10.74485 | 10.9662 | 8.73E-25 | 3.58E-23 | 45.37971 |
| TLR10 | 0.650308 | 4.498683 | 10.95987 | 9.22E-25 | 3.78E-23 | 45.32579 |
| RCSD1 | 0.695904 | 6.018232 | 10.95849 | 9.33E-25 | 3.81E-23 | 45.31407 |
| CARD11 | 0.642532 | 5.560021 | 10.90207 | 1.51E-24 | 6.15E-23 | 44.83467 |
| PIK3AP1 | 0.671868 | 5.36858 | 10.82372 | 2.96E-24 | 1.19E-22 | 44.17116 |
| TYMP | 0.924136 | 10.17057 | 10.79754 | 3.70E-24 | 1.48E-22 | 43.95004 |
| LYSMD2 | 0.674371 | 7.137374 | 10.78992 | 3.95E-24 | 1.57E-22 | 43.88576 |
| OAS2 | 0.931643 | 6.312501 | 10.76307 | 4.97E-24 | 1.97E-22 | 43.65934 |
| FKBP11 | 0.773993 | 7.887283 | 10.76174 | 5.02E-24 | 1.99E-22 | 43.64815 |
| IGLL1 | 1.18241 | 7.242378 | 10.74707 | 5.69E-24 | 2.24E-22 | 43.52465 |
| CAMK1G | 0.867738 | 5.043405 | 10.74135 | 5.97E-24 | 2.35E-22 | 43.47649 |
| SNX10 | 0.793571 | 6.15247 | 10.72425 | 6.91E-24 | 2.71E-22 | 43.3327 |
| IRF4 | 0.753841 | 4.819241 | 10.71847 | 7.26E-24 | 2.84E-22 | 43.28411 |
| ETV7 | 0.760606 | 5.380733 | 10.69033 | 9.21E-24 | 3.60E-22 | 43.04774 |
| CCND2 | 0.882703 | 7.722673 | 10.65201 | 1.27E-23 | 4.97E-22 | 42.72651 |
| IRF7 | 0.906255 | 7.7471 | 10.62054 | 1.66E-23 | 6.47E-22 | 42.46314 |
| SH2B3 | 0.621426 | 7.246347 | 10.61862 | 1.69E-23 | 6.56E-22 | 42.44709 |
| FCGR1B | 0.75281 | 5.445991 | 10.61786 | 1.70E-23 | 6.58E-22 | 42.44075 |
| IFI44L | 1.310681 | 7.526355 | 10.59806 | 2.01E-23 | 7.74E-22 | 42.27536 |
| IFI44 | 1.076976 | 8.293285 | 10.59073 | 2.14E-23 | 8.22E-22 | 42.2141 |
| TGM2 | 0.875806 | 7.276136 | 10.58864 | 2.18E-23 | 8.34E-22 | 42.19668 |
| ARHGDIB | 0.699455 | 11.05138 | 10.53485 | 3.43E-23 | 1.30E-21 | 41.74836 |
| RAB37 | 0.628479 | 4.712763 | 10.50908 | 4.25E-23 | 1.60E-21 | 41.53411 |
| CCL8 | 1.238443 | 7.668172 | 10.45875 | 6.49E-23 | 2.43E-21 | 41.11642 |
| ARHGEF3 | 0.593218 | 7.773986 | 10.44975 | 7.00E-23 | 2.61E-21 | 41.04192 |
| PIP4K2A | 0.599756 | 8.348313 | 10.43213 | 8.11E-23 | 3.02E-21 | 40.89601 |
| FAM30A | 0.601758 | 4.334059 | 10.38355 | 1.22E-22 | 4.51E-21 | 40.4946 |
| CPVL | 0.930154 | 7.567572 | 10.38172 | 1.24E-22 | 4.57E-21 | 40.47957 |
| CCL2 | 0.96922 | 8.448785 | 10.36887 | 1.38E-22 | 5.08E-21 | 40.37356 |
| IFIH1 | 0.928896 | 7.597881 | 10.34136 | 1.73E-22 | 6.36E-21 | 40.14689 |
| TCN2 | 0.647096 | 6.545382 | 10.33248 | 1.86E-22 | 6.83E-21 | 40.07384 |
| DPYD | 0.688908 | 5.513184 | 10.25681 | 3.49E-22 | 1.27E-20 | 39.45261 |
| LGALS2 | 0.647766 | 5.18264 | 10.25325 | 3.59E-22 | 1.31E-20 | 39.42352 |
| GPR84 | 0.68614 | 5.233377 | 10.2517 | 3.64E-22 | 1.32E-20 | 39.41084 |
| KLRG1 | 0.607957 | 5.152065 | 10.23273 | 4.26E-22 | 1.54E-20 | 39.25551 |
| CXCR5 | 0.693239 | 4.394804 | 10.16742 | 7.30E-22 | 2.64E-20 | 38.72238 |
| ADAM8 | 0.707937 | 5.989124 | 10.14319 | 8.92E-22 | 3.20E-20 | 38.52507 |
| IFITM1 | 1.024417 | 10.28117 | 10.09923 | 1.28E-21 | 4.59E-20 | 38.16785 |
| IFIT3 | 1.025166 | 7.436753 | 10.09463 | 1.33E-21 | 4.75E-20 | 38.13054 |
| PRKCH | 0.636172 | 6.982701 | 10.07967 | 1.50E-21 | 5.35E-20 | 38.00922 |
| PLCG2 | 0.852485 | 7.329201 | 10.07324 | 1.58E-21 | 5.63E-20 | 37.95707 |
| TLR1 | 0.67512 | 5.902149 | 10.05007 | 1.91E-21 | 6.74E-20 | 37.76951 |
| LGALS3BP | 1.010156 | 9.091043 | 10.04942 | 1.92E-21 | 6.77E-20 | 37.76422 |
| OASL | 1.044418 | 6.220293 | 10.03895 | 2.10E-21 | 7.35E-20 | 37.67956 |
| HSD11B1 | 0.691473 | 5.245462 | 10.02214 | 2.41E-21 | 8.42E-20 | 37.54367 |
| FNBP1 | 0.619098 | 8.27374 | 10.01797 | 2.49E-21 | 8.69E-20 | 37.51002 |
| FCRL5 | 0.58517 | 4.548377 | 9.981695 | 3.35E-21 | 1.16E-19 | 37.21743 |
| TMEM176A | 0.795094 | 6.721745 | 9.981299 | 3.36E-21 | 1.16E-19 | 37.21424 |
| TCIRG1 | 0.637383 | 7.364261 | 9.97957 | 3.41E-21 | 1.18E-19 | 37.20031 |
| CXCL11 | 1.199545 | 5.851792 | 9.958876 | 4.03E-21 | 1.39E-19 | 37.0337 |
| CCL19 | 1.79673 | 7.58288 | 9.9091 | 6.04E-21 | 2.07E-19 | 36.63383 |
| RUNX3 | 0.938872 | 7.538115 | 9.90444 | 6.27E-21 | 2.15E-19 | 36.59646 |
| RENBP | 0.719658 | 6.972029 | 9.884442 | 7.38E-21 | 2.52E-19 | 36.43621 |
| IKZF3 | 0.598909 | 5.009195 | 9.881911 | 7.53E-21 | 2.56E-19 | 36.41594 |
| ACP5 | 0.889748 | 6.825675 | 9.854954 | 9.37E-21 | 3.18E-19 | 36.20027 |
| TOX2 | 0.784212 | 5.932933 | 9.83203 | 1.13E-20 | 3.81E-19 | 36.01716 |
| PTGDS | 1.37496 | 6.860561 | 9.821358 | 1.23E-20 | 4.13E-19 | 35.93201 |
| HK3 | 0.740801 | 5.40873 | 9.816171 | 1.28E-20 | 4.29E-19 | 35.89063 |
| GLRX | 0.660611 | 7.255554 | 9.804624 | 1.41E-20 | 4.68E-19 | 35.7986 |
| PECAM1 | 0.661574 | 7.40603 | 9.782557 | 1.68E-20 | 5.58E-19 | 35.62288 |
| NR1H3 | 0.621334 | 7.386783 | 9.767494 | 1.90E-20 | 6.28E-19 | 35.50309 |
| EIF4E3 | 0.621904 | 6.192343 | 9.734238 | 2.48E-20 | 8.19E-19 | 35.23902 |
| BIRC3 | 0.762318 | 5.418306 | 9.694378 | 3.41E-20 | 1.12E-18 | 34.92325 |
| EMILIN2 | 0.686198 | 6.656736 | 9.688489 | 3.58E-20 | 1.17E-18 | 34.87668 |
| ITM2A | 1.096809 | 7.586598 | 9.686877 | 3.62E-20 | 1.18E-18 | 34.86393 |
| TNFRSF13B | 0.644986 | 4.45834 | 9.673941 | 4.02E-20 | 1.31E-18 | 34.76168 |
| DHX58 | 0.631172 | 6.285725 | 9.667579 | 4.23E-20 | 1.37E-18 | 34.71142 |
| CTSH | 0.61908 | 9.540661 | 9.630963 | 5.66E-20 | 1.83E-18 | 34.42259 |
| C3 | 1.137063 | 8.174032 | 9.628456 | 5.78E-20 | 1.86E-18 | 34.40284 |
| TIMD4 | 0.597939 | 4.386554 | 9.625866 | 5.90E-20 | 1.90E-18 | 34.38245 |
| CD8B | 0.632613 | 4.751953 | 9.62179 | 6.09E-20 | 1.95E-18 | 34.35034 |
| SIT1 | 0.588454 | 4.849933 | 9.598628 | 7.33E-20 | 2.35E-18 | 34.16811 |
| HLA-DQB1 | 1.1778 | 7.174849 | 9.589677 | 7.87E-20 | 2.51E-18 | 34.09776 |
| GALM | 0.682594 | 6.462404 | 9.57907 | 8.56E-20 | 2.72E-18 | 34.01446 |
| MICA | 0.615015 | 6.002104 | 9.562643 | 9.75E-20 | 3.10E-18 | 33.88555 |
| REC8 | 0.594171 | 5.740607 | 9.560732 | 9.90E-20 | 3.14E-18 | 33.87056 |
| FBXO6 | 0.602614 | 6.962496 | 9.538 | 1.19E-19 | 3.75E-18 | 33.69244 |
| IL2RA | 0.665147 | 4.996198 | 9.534595 | 1.22E-19 | 3.85E-18 | 33.66579 |
| BST2 | 1.133638 | 8.348771 | 9.531672 | 1.25E-19 | 3.92E-18 | 33.64291 |
| EAF2 | 0.654939 | 5.707435 | 9.52267 | 1.34E-19 | 4.19E-18 | 33.57247 |
| HLA-A | 1.706965 | 9.669976 | 9.495219 | 1.66E-19 | 5.19E-18 | 33.35796 |
| PARP10 | 0.731629 | 7.292611 | 9.453886 | 2.31E-19 | 7.15E-18 | 33.03573 |
| FPR1 | 0.692584 | 5.592634 | 9.452021 | 2.34E-19 | 7.23E-18 | 33.02121 |
| MAL | 0.966479 | 5.399579 | 9.443516 | 2.50E-19 | 7.70E-18 | 32.95502 |
| PLA2G7 | 0.870009 | 6.720546 | 9.424989 | 2.90E-19 | 8.88E-18 | 32.81098 |
| APOL1 | 0.6158 | 5.96247 | 9.388041 | 3.87E-19 | 1.18E-17 | 32.52428 |
| CCL13 | 1.05716 | 5.709924 | 9.367284 | 4.56E-19 | 1.39E-17 | 32.36353 |
| PLA1A | 0.615554 | 4.84783 | 9.355164 | 5.01E-19 | 1.52E-17 | 32.26978 |
| C15orf48 | 0.982102 | 7.429868 | 9.351981 | 5.14E-19 | 1.56E-17 | 32.24517 |
| GAPT | 0.659181 | 5.231966 | 9.331453 | 6.03E-19 | 1.82E-17 | 32.08659 |
| CD83 | 0.654757 | 7.415629 | 9.33068 | 6.07E-19 | 1.83E-17 | 32.08063 |
| GSDMD | 0.612429 | 7.576638 | 9.307306 | 7.28E-19 | 2.19E-17 | 31.90036 |
| RGS1 | 0.960525 | 7.722212 | 9.288667 | 8.42E-19 | 2.53E-17 | 31.75682 |
| SUSD3 | 0.812661 | 6.11588 | 9.248218 | 1.15E-18 | 3.45E-17 | 31.44597 |
| OAS1 | 0.849938 | 6.642222 | 9.108259 | 3.41E-18 | 1.00E-16 | 30.37736 |
| ICAM2 | 0.612832 | 6.767923 | 9.090408 | 3.91E-18 | 1.14E-16 | 30.24185 |
| FCGR2A | 0.692034 | 6.974452 | 9.07752 | 4.32E-18 | 1.25E-16 | 30.14413 |
| IFI27 | 1.085693 | 10.29613 | 9.075001 | 4.40E-18 | 1.28E-16 | 30.12504 |
| MSN | 0.588615 | 9.788337 | 9.033848 | 6.03E-18 | 1.74E-16 | 29.81367 |
| MS4A4A | 0.621085 | 5.59667 | 8.972537 | 9.64E-18 | 2.76E-16 | 29.35155 |
| STAT5A | 0.6279 | 6.798214 | 8.966509 | 1.01E-17 | 2.88E-16 | 29.30623 |
| FAM20A | 0.6185 | 5.287541 | 8.937209 | 1.26E-17 | 3.59E-16 | 29.08624 |
| SOD2 | 0.869869 | 8.649711 | 8.924166 | 1.39E-17 | 3.94E-16 | 28.98847 |
| LYN | 0.691311 | 8.310757 | 8.91086 | 1.54E-17 | 4.35E-16 | 28.88883 |
| CDK12 | 0.713026 | 6.724688 | 8.907021 | 1.59E-17 | 4.47E-16 | 28.8601 |
| TCL1A | 0.795596 | 4.5405 | 8.904125 | 1.62E-17 | 4.55E-16 | 28.83843 |
| CFB | 1.259217 | 7.472122 | 8.873438 | 2.05E-17 | 5.71E-16 | 28.60914 |
| BANK1 | 0.794782 | 4.868105 | 8.813394 | 3.22E-17 | 8.94E-16 | 28.16205 |
| SERPINA1 | 0.970261 | 7.659445 | 8.783125 | 4.04E-17 | 1.12E-15 | 27.93747 |
| CLEC7A | 0.624589 | 5.334178 | 8.77181 | 4.40E-17 | 1.21E-15 | 27.85364 |
| CD1B | 0.594888 | 4.432948 | 8.741157 | 5.54E-17 | 1.52E-15 | 27.62695 |
| RASSF2 | 0.696803 | 7.70414 | 8.705739 | 7.22E-17 | 1.96E-15 | 27.3657 |
| IFIT2 | 0.903782 | 7.364169 | 8.657438 | 1.04E-16 | 2.80E-15 | 27.01061 |
| PARP12 | 0.59389 | 7.525958 | 8.636978 | 1.21E-16 | 3.23E-15 | 26.86061 |
| LIME1 | 0.606948 | 6.473552 | 8.593396 | 1.67E-16 | 4.42E-15 | 26.54193 |
| BCL11B | 0.7367 | 5.637021 | 8.537453 | 2.52E-16 | 6.65E-15 | 26.13451 |
| LGMN | 0.655898 | 8.364158 | 8.519191 | 2.88E-16 | 7.57E-15 | 26.00192 |
| MX2 | 0.740412 | 7.069696 | 8.484491 | 3.72E-16 | 9.68E-15 | 25.75053 |
| C1R | 0.815533 | 7.466326 | 8.467265 | 4.22E-16 | 1.09E-14 | 25.626 |
| LPAR6 | 0.586716 | 6.804428 | 8.464246 | 4.32E-16 | 1.12E-14 | 25.6042 |
| EGFL6 | 0.839092 | 5.979692 | 8.384617 | 7.72E-16 | 1.98E-14 | 25.03104 |
| ANKRD22 | 0.720917 | 5.97969 | 8.381008 | 7.93E-16 | 2.03E-14 | 25.00515 |
| MX1 | 1.051497 | 9.875522 | 8.379716 | 8.00E-16 | 2.04E-14 | 24.99589 |
| GYPC | 0.746649 | 7.175815 | 8.367085 | 8.77E-16 | 2.23E-14 | 24.90536 |
| SLC2A6 | 0.685719 | 6.105111 | 8.360932 | 9.18E-16 | 2.32E-14 | 24.8613 |
| FUCA1 | 0.588875 | 8.146433 | 8.31413 | 1.29E-15 | 3.25E-14 | 24.52691 |
| SLC2A5 | 0.660716 | 5.905516 | 8.252488 | 2.01E-15 | 5.02E-14 | 24.08854 |
| CCL22 | 0.686231 | 5.188276 | 8.202281 | 2.89E-15 | 7.14E-14 | 23.73324 |
| SLC1A3 | 0.689308 | 6.644516 | 8.132527 | 4.75E-15 | 1.16E-13 | 23.2422 |
| ERAP2 | 0.89836 | 6.114856 | 8.106073 | 5.74E-15 | 1.40E-13 | 23.05678 |
| CD1C | 0.671524 | 5.057313 | 8.041428 | 9.08E-15 | 2.19E-13 | 22.60552 |
| DHRS9 | 0.746748 | 5.084886 | 8.030143 | 9.83E-15 | 2.36E-13 | 22.52702 |
| VSIG4 | 0.709613 | 6.174234 | 8.029337 | 9.89E-15 | 2.37E-13 | 22.52141 |
| ITM2C | 0.720538 | 8.66508 | 8.012758 | 1.11E-14 | 2.66E-13 | 22.40623 |
| FOLR2 | 0.759244 | 6.024746 | 8.007742 | 1.15E-14 | 2.75E-13 | 22.37142 |
| SLC2A3 | 0.650781 | 7.955588 | 7.947472 | 1.76E-14 | 4.18E-13 | 21.95438 |
| S100A4 | 0.770774 | 10.1302 | 7.899787 | 2.46E-14 | 5.80E-13 | 21.62607 |
| ETS1 | 0.669705 | 7.580761 | 7.887815 | 2.67E-14 | 6.28E-13 | 21.54388 |
| ARHGEF6 | 0.588772 | 7.684403 | 7.886756 | 2.69E-14 | 6.32E-13 | 21.53661 |
| ENPP2 | 0.830509 | 6.420338 | 7.835649 | 3.84E-14 | 8.91E-13 | 21.1868 |
| ABI3BP | 0.750958 | 5.504837 | 7.786886 | 5.39E-14 | 1.24E-12 | 20.85461 |
| BIN1 | 0.625135 | 6.940763 | 7.711382 | 9.06E-14 | 2.06E-12 | 20.34332 |
| CYFIP2 | 0.703995 | 6.992889 | 7.692942 | 1.03E-13 | 2.33E-12 | 20.21902 |
| RSAD2 | 0.796711 | 6.276695 | 7.692379 | 1.03E-13 | 2.34E-12 | 20.21522 |
| EPB41L3 | 0.626727 | 6.243142 | 7.649146 | 1.39E-13 | 3.12E-12 | 19.92469 |
| VPREB3 | 0.778717 | 5.280555 | 7.528955 | 3.14E-13 | 6.94E-12 | 19.1235 |
| IGHV5-78 | 0.599725 | 4.66809 | 7.492668 | 4.01E-13 | 8.81E-12 | 18.88351 |
| ALDH2 | 0.621848 | 7.386588 | 7.391778 | 7.87E-13 | 1.69E-11 | 18.22093 |
| MMP9 | 1.006064 | 9.127898 | 7.369132 | 9.15E-13 | 1.96E-11 | 18.07315 |
| CCL18 | 0.797321 | 5.468437 | 7.358052 | 9.85E-13 | 2.10E-11 | 18.00098 |
| MATK | 0.609883 | 5.51943 | 7.323721 | 1.24E-12 | 2.60E-11 | 17.77788 |
| TGFBR2 | 0.631577 | 8.112543 | 7.279635 | 1.65E-12 | 3.46E-11 | 17.49257 |
| BTG2 | 0.59813 | 7.701666 | 7.152197 | 3.81E-12 | 7.74E-11 | 16.67538 |
| GPR34 | 0.587576 | 5.490179 | 7.150434 | 3.85E-12 | 7.81E-11 | 16.66415 |
| TNFSF10 | 0.763991 | 8.52926 | 7.103075 | 5.23E-12 | 1.05E-10 | 16.3634 |
| MS4A7 | 0.585623 | 6.436043 | 7.094439 | 5.53E-12 | 1.11E-10 | 16.30873 |
| TDO2 | 0.639189 | 5.484523 | 7.075585 | 6.25E-12 | 1.25E-10 | 16.18955 |
| PYCARD | 0.664381 | 8.274753 | 7.050264 | 7.36E-12 | 1.46E-10 | 16.02987 |
| PRKCQ-AS1 | 0.613857 | 5.154468 | 6.910118 | 1.80E-11 | 3.46E-10 | 15.15434 |
| SPIB | 0.892612 | 5.049941 | 6.868415 | 2.34E-11 | 4.45E-10 | 14.89651 |
| PARM1 | 0.593965 | 6.421812 | 6.866805 | 2.36E-11 | 4.49E-10 | 14.88657 |
| RNF126P1 | 0.773423 | 4.744125 | 6.862873 | 2.42E-11 | 4.59E-10 | 14.86233 |
| ERBB3 | -0.61746 | 8.028756 | -6.84006 | 2.80E-11 | 5.26E-10 | 14.72192 |
| TUBB2A | -0.58869 | 6.469164 | -6.81688 | 3.24E-11 | 6.06E-10 | 14.5796 |
| RBP5 | 0.63893 | 5.48717 | 6.792072 | 3.78E-11 | 7.03E-10 | 14.42774 |
| ALDH1A1 | 0.697838 | 6.511388 | 6.74815 | 4.97E-11 | 9.15E-10 | 14.15994 |
| ST6GAL1 | 0.644186 | 7.961912 | 6.709346 | 6.32E-11 | 1.16E-09 | 13.92452 |
| HLA-DRB1 | 1.386 | 7.789086 | 6.662066 | 8.46E-11 | 1.53E-09 | 13.63915 |
| FBP1 | 0.790407 | 7.228728 | 6.589117 | 1.32E-10 | 2.37E-09 | 13.20206 |
| TNF | 0.592343 | 5.433493 | 6.558753 | 1.59E-10 | 2.84E-09 | 13.02128 |
| ISG15 | 0.858816 | 9.217962 | 6.535092 | 1.84E-10 | 3.25E-09 | 12.88088 |
| IFI6 | 0.844473 | 9.470004 | 6.492264 | 2.38E-10 | 4.19E-09 | 12.6278 |
| TMEM119 | 0.651968 | 7.562113 | 6.392634 | 4.33E-10 | 7.41E-09 | 12.04433 |
| MARCO | 0.8827 | 5.978857 | 6.292554 | 7.84E-10 | 1.31E-08 | 11.46571 |
| CR2 | 0.635106 | 4.626119 | 6.281783 | 8.35E-10 | 1.40E-08 | 11.40388 |
| PRR36 | -0.75253 | 6.605223 | -6.26869 | 9.02E-10 | 1.51E-08 | 11.32888 |
| RARRES1 | 1.019599 | 9.295485 | 6.219274 | 1.20E-09 | 2.00E-08 | 11.04683 |
| KLHDC7B | 0.684108 | 5.087539 | 6.111483 | 2.25E-09 | 3.61E-08 | 10.4381 |
| IFIT1 | 0.752472 | 7.869506 | 5.856577 | 9.53E-09 | 1.46E-07 | 9.0342 |
| SERTAD4 | -0.62514 | 7.513561 | -5.81233 | 1.22E-08 | 1.85E-07 | 8.79568 |
| CXCL12 | 0.636735 | 7.915002 | 5.606509 | 3.74E-08 | 5.44E-07 | 7.706424 |
| ALPL | 0.600615 | 7.216505 | 5.220371 | 2.81E-07 | 3.70E-06 | 5.754745 |
| F13A1 | 0.639146 | 6.227543 | 5.192432 | 3.24E-07 | 4.24E-06 | 5.618254 |
| CYP1B1 | 0.745959 | 7.804107 | 5.152973 | 3.95E-07 | 5.10E-06 | 5.426582 |
| GSTO2 | -0.58645 | 6.337386 | -5.05143 | 6.54E-07 | 8.17E-06 | 4.939275 |
| CCL21 | 0.817125 | 6.091524 | 5.03345 | 7.15E-07 | 8.89E-06 | 4.853875 |
| MMP12 | 0.799686 | 6.250972 | 4.848107 | 1.76E-06 | 2.04E-05 | 3.989403 |
| PIGR | 0.801428 | 5.273488 | 4.738987 | 2.94E-06 | 3.32E-05 | 3.494015 |
| AZGP1 | -0.87934 | 8.589567 | -4.48469 | 9.43E-06 | 9.80E-05 | 2.379018 |
| SAA1 | 0.733106 | 7.678071 | 4.470596 | 1.00E-05 | 0.000104 | 2.318856 |
| TTYH1 | -0.8169 | 6.129823 | -4.2948 | 2.17E-05 | 0.00021 | 1.582893 |
| BNIPL | -0.58711 | 6.500142 | -4.25178 | 2.61E-05 | 0.000249 | 1.40692 |
| KIAA1324 | -0.66522 | 7.78413 | -3.87167 | 0.000125 | 0.001005 | -0.07689 |
| KANK4 | -0.60526 | 6.556255 | -3.81306 | 0.000158 | 0.001231 | -0.29423 |
| PPP1R1B | -0.76496 | 7.593353 | -3.81076 | 0.000159 | 0.001241 | -0.30268 |
| S100A9 | 0.813218 | 9.763571 | 3.78591 | 0.000175 | 0.001353 | -0.39384 |
| SERPINB5 | -0.66783 | 6.902958 | -3.75631 | 0.000197 | 0.001493 | -0.5017 |
| S100A8 | 0.792997 | 8.907468 | 3.569224 | 0.000399 | 0.002783 | -1.165 |
| EEF1A2 | -0.63754 | 5.576513 | -3.52206 | 0.000475 | 0.003223 | -1.32719 |
| LTF | 0.806605 | 7.544176 | 3.488647 | 0.000537 | 0.003568 | -1.44086 |
| MMP7 | 0.794051 | 9.7007 | 3.481512 | 0.000551 | 0.003648 | -1.465 |
| COL2A1 | -0.6536 | 5.066931 | -3.42303 | 0.00068 | 0.00438 | -1.6611 |
| ORM1 | 0.708257 | 5.366945 | 3.351867 | 0.000875 | 0.005443 | -1.89552 |
| SCRG1 | -0.6134 | 5.782632 | -3.20655 | 0.001446 | 0.008336 | -2.35963 |
| SPDEF | -0.62499 | 6.518419 | -3.12615 | 0.001894 | 0.010431 | -2.608 |
| CALML5 | -0.67223 | 8.019503 | -2.71765 | 0.006846 | 0.030326 | -3.77646 |

FC:Fold change;
