# Supplementary material for: Immune checkpoints expression patterns in early-stage triple-negative breast cancer predict prognosis and remodel the tumor immune microenvironment
Source: Front Immunol. 2023 Feb 6;14:1073550. doi: 10.3389/fimmu.2023.1073550 (PMC9939840; doi:10.3389/fimmu.2023.1073550)
Supplement: Supplementary file 11 [file Table_4.docx]

| Table. S4 The results of Cox regression analysis for DEGs between different ICGs clusters. | | | | |
| --- | --- | --- | --- | --- |
| id | HR | HR.95L | HR.95H | pvalue |
| APOBEC3G | 0.799921 | 0.686161 | 0.932543 | 0.00434 |
| GBP4 | 0.826445 | 0.739095 | 0.924119 | 0.000824 |
| HLA-DMA | 0.830248 | 0.719829 | 0.957604 | 0.010621 |
| IRF1 | 0.798618 | 0.694273 | 0.918645 | 0.001645 |
| HLA-E | 0.830965 | 0.696811 | 0.990946 | 0.039283 |
| HLA-DMB | 0.830632 | 0.722643 | 0.954758 | 0.009015 |
| GZMA | 0.844759 | 0.750532 | 0.950817 | 0.005178 |
| CD2 | 0.846574 | 0.757063 | 0.946667 | 0.003486 |
| IL18BP | 0.832042 | 0.706388 | 0.980048 | 0.027719 |
| CXCR3 | 0.811015 | 0.692347 | 0.950021 | 0.009454 |
| CCL5 | 0.855939 | 0.775252 | 0.945025 | 0.002075 |
| PSMB10 | 0.769976 | 0.637456 | 0.930046 | 0.006677 |
| HLA-B | 0.820318 | 0.714936 | 0.941233 | 0.004753 |
| GBP5 | 0.839777 | 0.757726 | 0.930712 | 0.000872 |
| CD3D | 0.836625 | 0.748605 | 0.934994 | 0.001661 |
| PSMB9 | 0.849147 | 0.74737 | 0.964785 | 0.012062 |
| CD53 | 0.836257 | 0.709085 | 0.986238 | 0.033619 |
| HLA-DRA | 0.841892 | 0.749449 | 0.945737 | 0.003731 |
| NKG7 | 0.895671 | 0.803607 | 0.998282 | 0.046476 |
| HLA-DQA1 | 0.869346 | 0.784589 | 0.963259 | 0.007469 |
| HLA-DPA1 | 0.840037 | 0.747207 | 0.944399 | 0.003529 |
| HLA-F | 0.800901 | 0.698084 | 0.918861 | 0.00154 |
| HCST | 0.829116 | 0.722489 | 0.951478 | 0.007627 |
| ARHGAP9 | 0.821661 | 0.700641 | 0.963585 | 0.015681 |
| IDO1 | 0.845237 | 0.768624 | 0.929485 | 0.000524 |
| PLEK | 0.841789 | 0.737181 | 0.961242 | 0.010965 |
| BTN3A3 | 0.7335 | 0.602858 | 0.892455 | 0.001956 |
| CXCR6 | 0.723309 | 0.594215 | 0.880449 | 0.001241 |
| ICOS | 0.758566 | 0.640875 | 0.89787 | 0.001317 |
| GIMAP4 | 0.844683 | 0.726256 | 0.982422 | 0.02852 |
| CD8A | 0.830216 | 0.733186 | 0.940088 | 0.003344 |
| PRF1 | 0.824478 | 0.706255 | 0.962492 | 0.014522 |
| CD247 | 0.809392 | 0.708595 | 0.924527 | 0.001831 |
| HLA-DOA | 0.843122 | 0.739622 | 0.961106 | 0.010661 |
| CYBB | 0.868197 | 0.760187 | 0.991554 | 0.037061 |
| HCLS1 | 0.817674 | 0.706016 | 0.946991 | 0.007209 |
| CST7 | 0.835715 | 0.715457 | 0.976187 | 0.023574 |
| RAC2 | 0.847509 | 0.743574 | 0.965971 | 0.01319 |
| SLAMF1 | 0.776382 | 0.636059 | 0.947661 | 0.012828 |
| EVI2B | 0.860907 | 0.749961 | 0.988266 | 0.033366 |
| FGL2 | 0.865349 | 0.759523 | 0.985919 | 0.029777 |
| IRF8 | 0.830709 | 0.729472 | 0.945996 | 0.005154 |
| GZMB | 0.842571 | 0.759995 | 0.93412 | 0.001134 |
| BTN3A1 | 0.755751 | 0.62173 | 0.918661 | 0.004927 |
| IL10RA | 0.83165 | 0.706979 | 0.978306 | 0.026105 |
| CORO1A | 0.798557 | 0.684888 | 0.931093 | 0.004088 |
| SASH3 | 0.83619 | 0.711852 | 0.982246 | 0.029402 |
| CD48 | 0.842756 | 0.739078 | 0.960979 | 0.010642 |
| PYHIN1 | 0.774349 | 0.635905 | 0.942935 | 0.010939 |
| TNFRSF1B | 0.78146 | 0.64229 | 0.950784 | 0.013727 |
| ARHGAP25 | 0.82831 | 0.693754 | 0.988964 | 0.037282 |
| SAMSN1 | 0.825312 | 0.683416 | 0.996669 | 0.046083 |
| CD7 | 0.821873 | 0.708017 | 0.954038 | 0.009926 |
| MAP4K1 | 0.855951 | 0.734072 | 0.998067 | 0.047183 |
| CXCL9 | 0.877542 | 0.814707 | 0.945223 | 0.000569 |
| RASAL3 | 0.839282 | 0.719468 | 0.97905 | 0.025789 |
| LTA | 0.80135 | 0.659829 | 0.973225 | 0.025502 |
| IFNG | 0.780001 | 0.648847 | 0.937665 | 0.008165 |
| CYTIP | 0.804093 | 0.6674 | 0.968784 | 0.021816 |
| PSTPIP1 | 0.807903 | 0.65308 | 0.999429 | 0.049388 |
| CD38 | 0.828477 | 0.725892 | 0.945558 | 0.005271 |
| GPSM3 | 0.79823 | 0.66446 | 0.958931 | 0.016037 |
| DOCK2 | 0.849392 | 0.728321 | 0.990589 | 0.037481 |
| ITK | 0.800499 | 0.678253 | 0.944777 | 0.008493 |
| CD3G | 0.79093 | 0.659502 | 0.948549 | 0.011417 |
| DOCK10 | 0.800382 | 0.668644 | 0.958077 | 0.015236 |
| STX11 | 0.785904 | 0.651449 | 0.94811 | 0.011851 |
| TAP1 | 0.837695 | 0.743861 | 0.943365 | 0.00348 |
| SLA | 0.799452 | 0.65208 | 0.980131 | 0.031318 |
| GBP1 | 0.816051 | 0.723157 | 0.920878 | 0.000978 |
| PRKCB | 0.814846 | 0.707605 | 0.93834 | 0.004456 |
| ZBED2 | 0.716348 | 0.575699 | 0.891359 | 0.002779 |
| LPXN | 0.808646 | 0.677407 | 0.965311 | 0.018738 |
| IL18RAP | 0.605364 | 0.464898 | 0.78827 | 0.000194 |
| GZMK | 0.870754 | 0.784646 | 0.966311 | 0.009187 |
| GNLY | 0.837997 | 0.750607 | 0.935561 | 0.001659 |
| ARHGAP30 | 0.772578 | 0.639149 | 0.933862 | 0.007646 |
| AIM2 | 0.837358 | 0.728101 | 0.963008 | 0.012833 |
| CTLA4 | 0.733048 | 0.615367 | 0.873233 | 0.000505 |
| STAT4 | 0.748664 | 0.623681 | 0.898693 | 0.001895 |
| FASLG | 0.787414 | 0.624798 | 0.992353 | 0.042867 |
| ZNF683 | 0.815035 | 0.695944 | 0.954506 | 0.011159 |
| NLRC5 | 0.745806 | 0.61391 | 0.906039 | 0.00314 |
| GMFG | 0.804453 | 0.676504 | 0.956602 | 0.013817 |
| CXCL13 | 0.849843 | 0.774866 | 0.932075 | 0.000555 |
| TBC1D10C | 0.810957 | 0.697181 | 0.9433 | 0.006592 |
| SAMD3 | 0.720805 | 0.555714 | 0.934941 | 0.01363 |
| GZMH | 0.833604 | 0.716632 | 0.969668 | 0.018312 |
| CTSW | 0.818502 | 0.691341 | 0.969052 | 0.020077 |
| SH2D1A | 0.758776 | 0.634958 | 0.906739 | 0.002389 |
| PDCD1 | 0.805726 | 0.661079 | 0.982023 | 0.032383 |
| CD6 | 0.81158 | 0.698869 | 0.942469 | 0.006207 |
| ACAP1 | 0.817537 | 0.6873 | 0.972453 | 0.022878 |
| APOBEC3F | 0.779505 | 0.637254 | 0.95351 | 0.015391 |
| GIMAP5 | 0.83133 | 0.693502 | 0.996551 | 0.04579 |
| CD27 | 0.825653 | 0.713909 | 0.954887 | 0.009819 |
| PSMB8 | 0.841086 | 0.723205 | 0.978183 | 0.024686 |
| UBD | 0.880788 | 0.8184 | 0.947933 | 0.000708 |
| IL2RG | 0.78987 | 0.656855 | 0.949822 | 0.012172 |
| IGSF6 | 0.844156 | 0.714846 | 0.996858 | 0.045818 |
| STAT1 | 0.869857 | 0.769429 | 0.983393 | 0.025913 |
| HLA-DPB1 | 0.842458 | 0.721242 | 0.984045 | 0.03055 |
| CSF2RA | 0.810524 | 0.674248 | 0.974344 | 0.025306 |
| GIMAP6 | 0.833482 | 0.705265 | 0.985009 | 0.032582 |
| LCP2 | 0.743775 | 0.579986 | 0.953817 | 0.019672 |
| BTN3A2 | 0.7991 | 0.675495 | 0.945322 | 0.008902 |
| GBP2 | 0.819035 | 0.712391 | 0.941644 | 0.005035 |
| CD40 | 0.778917 | 0.641747 | 0.945407 | 0.011472 |
| TLR8 | 0.83525 | 0.697664 | 0.999968 | 0.04996 |
| ARHGAP15 | 0.821609 | 0.689413 | 0.979152 | 0.028138 |
| HLA-DOB | 0.784193 | 0.673837 | 0.912621 | 0.00168 |
| PARVG | 0.80805 | 0.664227 | 0.983015 | 0.033067 |
| PSME2 | 0.740902 | 0.605089 | 0.907199 | 0.0037 |
| HLA-H | 0.833233 | 0.740123 | 0.938056 | 0.002547 |
| ZAP70 | 0.821308 | 0.683489 | 0.986916 | 0.035686 |
| CD52 | 0.859035 | 0.776243 | 0.950657 | 0.003297 |
| LY9 | 0.774046 | 0.607904 | 0.985594 | 0.03774 |
| IGFLR1 | 0.808005 | 0.660888 | 0.987872 | 0.037621 |
| P2RY10 | 0.804765 | 0.669616 | 0.967192 | 0.02058 |
| SLAMF7 | 0.801879 | 0.668833 | 0.961393 | 0.017065 |
| TNFRSF14 | 0.767534 | 0.635761 | 0.926619 | 0.005905 |
| PTPRC | 0.81645 | 0.688311 | 0.968443 | 0.019908 |
| GIMAP7 | 0.866233 | 0.753479 | 0.995859 | 0.043562 |
| P2RY8 | 0.827227 | 0.700021 | 0.977549 | 0.02598 |
| AKNA | 0.815412 | 0.678065 | 0.980579 | 0.030132 |
| SRGN | 0.855908 | 0.75889 | 0.965329 | 0.01125 |
| S1PR4 | 0.790165 | 0.638107 | 0.978458 | 0.030799 |
| TNFSF13B | 0.848778 | 0.74791 | 0.96325 | 0.011084 |
| PIM2 | 0.838811 | 0.732245 | 0.960885 | 0.011227 |
| ALOX5 | 0.834114 | 0.71262 | 0.976321 | 0.023925 |
| TRAT1 | 0.735227 | 0.589276 | 0.917327 | 0.006445 |
| IL7R | 0.855881 | 0.761346 | 0.962155 | 0.00916 |
| KLRB1 | 0.791043 | 0.677703 | 0.923338 | 0.00297 |
| UBASH3A | 0.736144 | 0.569106 | 0.95221 | 0.019653 |
| LAT2 | 0.791765 | 0.637701 | 0.983049 | 0.034446 |
| RASSF5 | 0.760106 | 0.641932 | 0.900035 | 0.001465 |
| CCR2 | 0.774474 | 0.623663 | 0.961753 | 0.020729 |
| WIPF1 | 0.78711 | 0.651289 | 0.951255 | 0.013247 |
| PSME1 | 0.77423 | 0.627041 | 0.95597 | 0.017383 |
| B2M | 0.822562 | 0.709392 | 0.953786 | 0.009696 |
| CD69 | 0.815313 | 0.700156 | 0.949409 | 0.008584 |
| CLEC4A | 0.796833 | 0.64749 | 0.98062 | 0.03197 |
| CXCL10 | 0.917181 | 0.847028 | 0.993145 | 0.033223 |
| TNFAIP3 | 0.821776 | 0.686409 | 0.98384 | 0.032568 |
| CLECL1 | 0.705965 | 0.560294 | 0.88951 | 0.003148 |
| SEMA4D | 0.766928 | 0.624937 | 0.94118 | 0.011077 |
| PTPN6 | 0.807922 | 0.657072 | 0.993403 | 0.0431 |
| HLA-G | 0.8207 | 0.705118 | 0.955227 | 0.010728 |
| THEMIS | 0.679896 | 0.514293 | 0.898823 | 0.00675 |
| RGS18 | 0.741536 | 0.573782 | 0.958335 | 0.022302 |
| ACSL5 | 0.837514 | 0.715092 | 0.980896 | 0.027864 |
| PTPN22 | 0.778306 | 0.612391 | 0.989171 | 0.040465 |
| P2RY13 | 0.775815 | 0.613103 | 0.98171 | 0.034543 |
| LTB | 0.853589 | 0.769463 | 0.946914 | 0.002787 |
| SELL | 0.846844 | 0.746197 | 0.961066 | 0.01002 |
| PLCB2 | 0.774264 | 0.61879 | 0.968801 | 0.025279 |
| KLRD1 | 0.638091 | 0.484438 | 0.840478 | 0.001392 |
| LCP1 | 0.8123 | 0.713123 | 0.92527 | 0.001754 |
| VCAM1 | 0.846408 | 0.74889 | 0.956625 | 0.007586 |
| BTLA | 0.728874 | 0.565525 | 0.939405 | 0.014574 |
| PRDM1 | 0.817583 | 0.674164 | 0.991512 | 0.040699 |
| NAPSB | 0.844419 | 0.739585 | 0.964114 | 0.012409 |
| GIMAP2 | 0.803049 | 0.647039 | 0.996674 | 0.04657 |
| CCR7 | 0.860718 | 0.756131 | 0.979773 | 0.023261 |
| IL15RA | 0.693617 | 0.551947 | 0.87165 | 0.001699 |
| IFI16 | 0.769663 | 0.654168 | 0.905549 | 0.0016 |
| PLAC8 | 0.835978 | 0.721051 | 0.969224 | 0.017585 |
| TRIM69 | 0.636325 | 0.478717 | 0.845823 | 0.001851 |
| RAB8B | 0.771726 | 0.623131 | 0.955756 | 0.017565 |
| IL23A | 0.727688 | 0.56038 | 0.944948 | 0.01709 |
| CTSC | 0.825576 | 0.710131 | 0.959788 | 0.012631 |
| CD79A | 0.876819 | 0.802027 | 0.958586 | 0.003855 |
| PAG1 | 0.686446 | 0.549208 | 0.857978 | 0.000947 |
| CD79B | 0.841919 | 0.740009 | 0.957864 | 0.008951 |
| PIK3CD | 0.795769 | 0.649999 | 0.97423 | 0.026907 |
| SEL1L3 | 0.840376 | 0.717442 | 0.984375 | 0.031152 |
| MEI1 | 0.744908 | 0.563614 | 0.984517 | 0.038487 |
| TRAF3IP3 | 0.778558 | 0.651738 | 0.930055 | 0.005793 |
| LAMP3 | 0.839905 | 0.748043 | 0.943049 | 0.003155 |
| ZNF831 | 0.747132 | 0.571651 | 0.976481 | 0.032826 |
| TNFRSF4 | 0.802307 | 0.646492 | 0.995676 | 0.045576 |
| TNFRSF17 | 0.858923 | 0.759411 | 0.971476 | 0.015496 |
| TMEM176B | 0.695343 | 0.57353 | 0.843027 | 0.000218 |
| DEF6 | 0.688674 | 0.541234 | 0.87628 | 0.00241 |
| TRAF1 | 0.735485 | 0.577189 | 0.937194 | 0.012972 |
| IGHG1 | 0.911838 | 0.858585 | 0.968394 | 0.002647 |
| TAPBP | 0.816016 | 0.667911 | 0.996962 | 0.046619 |
| NFS1 | 0.778064 | 0.654266 | 0.925287 | 0.004537 |
| PLCL2 | 0.721513 | 0.594973 | 0.874967 | 0.000908 |
| IRF9 | 0.763982 | 0.619655 | 0.941925 | 0.011735 |
| LRMP | 0.829838 | 0.691888 | 0.995292 | 0.044343 |
| IL15 | 0.789442 | 0.628452 | 0.991673 | 0.042171 |
| C1S | 0.836163 | 0.734208 | 0.952276 | 0.006996 |
| TAPBPL | 0.651971 | 0.518287 | 0.820138 | 0.000259 |
| IGKC | 0.881204 | 0.811262 | 0.957176 | 0.002724 |
| SPOCK2 | 0.83479 | 0.73946 | 0.942409 | 0.003515 |
| RTP4 | 0.839357 | 0.730481 | 0.96446 | 0.013494 |
| STK17B | 0.778376 | 0.626973 | 0.966339 | 0.023194 |
| PTGER4 | 0.740359 | 0.608103 | 0.901381 | 0.002753 |
| PVRIG | 0.787699 | 0.645026 | 0.961931 | 0.01925 |
| IL32 | 0.855495 | 0.747979 | 0.978466 | 0.022747 |
| CD274 | 0.724188 | 0.553247 | 0.947947 | 0.018819 |
| SKAP1 | 0.850041 | 0.729653 | 0.990292 | 0.037054 |
| CD19 | 0.850621 | 0.747224 | 0.968324 | 0.014415 |
| MZB1 | 0.893044 | 0.817035 | 0.976124 | 0.012688 |
| STAMBPL1 | 0.679835 | 0.547897 | 0.843545 | 0.000456 |
| TNFAIP8 | 0.754033 | 0.600132 | 0.947402 | 0.015357 |
| FGD3 | 0.732621 | 0.608321 | 0.882319 | 0.001039 |
| EFHD2 | 0.743521 | 0.594019 | 0.93065 | 0.009668 |
| GPR18 | 0.802837 | 0.652088 | 0.988434 | 0.03849 |
| PPP1R16B | 0.794413 | 0.676703 | 0.932598 | 0.004911 |
| CELF2 | 0.761358 | 0.614908 | 0.942687 | 0.012369 |
| IL18R1 | 0.633563 | 0.492244 | 0.815452 | 0.000394 |
| RFTN1 | 0.770886 | 0.641816 | 0.925911 | 0.00538 |
| CDC42SE2 | 0.765091 | 0.606046 | 0.965874 | 0.024323 |
| RCSD1 | 0.768286 | 0.615894 | 0.958383 | 0.01945 |
| LYSMD2 | 0.746713 | 0.614728 | 0.907037 | 0.003249 |
| IGLL1 | 0.84867 | 0.755778 | 0.952978 | 0.005532 |
| SNX10 | 0.788162 | 0.66008 | 0.941096 | 0.008515 |
| CCND2 | 0.743064 | 0.631382 | 0.8745 | 0.000352 |
| FAM30A | 0.759375 | 0.600644 | 0.960053 | 0.021408 |
| TCN2 | 0.795893 | 0.641774 | 0.987023 | 0.037626 |
| LGALS2 | 0.743259 | 0.595176 | 0.928187 | 0.008862 |
| KLRG1 | 0.792291 | 0.627875 | 0.999761 | 0.049765 |
| CXCR5 | 0.811547 | 0.659874 | 0.998083 | 0.047913 |
| PRKCH | 0.786253 | 0.638919 | 0.967563 | 0.023121 |
| PLCG2 | 0.835598 | 0.716123 | 0.975005 | 0.022519 |
| LGALS3BP | 0.838183 | 0.738479 | 0.951348 | 0.006298 |
| HSD11B1 | 0.793451 | 0.649653 | 0.969079 | 0.023339 |
| FNBP1 | 0.714164 | 0.575261 | 0.886607 | 0.002284 |
| FCRL5 | 0.777897 | 0.609378 | 0.993018 | 0.043778 |
| TMEM176A | 0.721313 | 0.610431 | 0.852336 | 0.000125 |
| CXCL11 | 0.842308 | 0.751577 | 0.943992 | 0.003166 |
| CCL19 | 0.922261 | 0.855984 | 0.993669 | 0.03343 |
| RUNX3 | 0.794691 | 0.690573 | 0.914507 | 0.00134 |
| TOX2 | 0.819974 | 0.689931 | 0.97453 | 0.024272 |
| PTGDS | 0.871292 | 0.788454 | 0.962833 | 0.006871 |
| GLRX | 0.798263 | 0.659968 | 0.965539 | 0.020273 |
| EIF4E3 | 0.796482 | 0.650071 | 0.975866 | 0.028114 |
| BIRC3 | 0.823034 | 0.687039 | 0.985948 | 0.034555 |
| ITM2A | 0.847534 | 0.752297 | 0.954828 | 0.006528 |
| CTSH | 0.735663 | 0.605723 | 0.893478 | 0.001963 |
| MICA | 0.791074 | 0.644376 | 0.97117 | 0.025123 |
| REC8 | 0.777192 | 0.625592 | 0.965529 | 0.022797 |
| IL2RA | 0.783321 | 0.638061 | 0.96165 | 0.019614 |
| HLA-A | 0.907413 | 0.84691 | 0.972239 | 0.005786 |
| MAL | 0.863541 | 0.749885 | 0.994423 | 0.041586 |
| CCL13 | 0.863807 | 0.760913 | 0.980614 | 0.023668 |
| CD83 | 0.737648 | 0.609626 | 0.892554 | 0.001756 |
| SUSD3 | 0.800974 | 0.681356 | 0.941591 | 0.007161 |
| FAM20A | 0.782391 | 0.636625 | 0.961532 | 0.019655 |
| BANK1 | 0.806808 | 0.681213 | 0.955558 | 0.0129 |
| CD1B | 0.742535 | 0.59388 | 0.928399 | 0.009007 |
| RASSF2 | 0.771164 | 0.650046 | 0.914849 | 0.002874 |
| BCL11B | 0.775194 | 0.654318 | 0.918401 | 0.003239 |
| LPAR6 | 0.820556 | 0.674871 | 0.997689 | 0.04735 |
| ANKRD22 | 0.830696 | 0.711096 | 0.970411 | 0.019353 |
| ETS1 | 0.827679 | 0.706362 | 0.969831 | 0.019347 |
| EPB41L3 | 0.837646 | 0.707471 | 0.991774 | 0.0398 |
| ALDH2 | 0.826132 | 0.699958 | 0.97505 | 0.023898 |
| MATK | 0.787422 | 0.659622 | 0.939982 | 0.00817 |
| PRKCQ-AS1 | 0.828216 | 0.705042 | 0.972909 | 0.021775 |
| PARM1 | 0.808696 | 0.686165 | 0.953108 | 0.011313 |
| TUBB2A | 1.16768 | 1.001775 | 1.36106 | 0.047407 |
| FBP1 | 0.886667 | 0.789416 | 0.9959 | 0.04243 |
| TNF | 0.839378 | 0.714701 | 0.985805 | 0.032825 |
| MARCO | 0.900863 | 0.814692 | 0.996149 | 0.041833 |
| AZGP1 | 1.095925 | 1.018233 | 1.179545 | 0.014622 |
| MMP7 | 0.927306 | 0.873633 | 0.984277 | 0.013104 |
| SPDEF | 1.095666 | 1.022909 | 1.173596 | 0.009159 |

HR:Hazard ratios; HR.95L: 95% lower confidence intervals; HR.95H: 95% higher confidence intervals;
